# Development of Zn_1−x_Sn_x_O and Mg_1−x_Sn_x_O transparent conducting oxide thin films for perovskite solar cell applications

**DOI:** 10.1038/s41598-026-42690-x

**Published:** 2026-04-03

**Authors:** G. Kiruthiga, M. Sathish Kumar, T. Raguram, Arun Prasad Murali, Chandini Ragumoorthy, Rakesh Kumar, Jayant Giri, Faruq Mohammad, Ahmed A. Soleiman, Islom Kadirov

**Affiliations:** 1 Department of Science and Humanities (Physics), FOE, Karpagam Academy of Higher Education, Coimbatore, Tamil Nadu 641 021 India; 2 Centre for Smart Energy Systems, Chennai Institute of Technology, Chennai, Tamil Nadu 600069 India; 3 Department of Chemical and Materials Engineering, Tunghai University, Taichung City, 40704 Taiwan; 4 Laboratory of Physical Chemistry and Solid-State Electrochemistry (LFES), Department of Materials Chemistry, Faculty of Chemistry and Biology, University of Santiago de Chile (USACH), Santiago, Chile; 5 Department of Mechanical Engineering, Vel Tech Rangarajan Dr. Sagunthala R&D Institute of Science and Technology, Chennai, India; 6 Department of Chemical Engineering and Biotechnology, National Taipei University of Technology, Taipei, Taiwan; 7 Department of Mechanical Engineering, Manipal University Jaipur, Jaipur, Rajasthan 303007 India; 8 Department of Mechanical Engineering, Yeshwantrao Chavan College of Engineering, Nagpur, India; 9 Division of Research and Development, Lovely Professional University, Phagwara, India; 10 Centre for Research Impact and Outcome, Chitkara University Institute of Engineering and Technology, Chitkara University, Rajpura, Punjab 140401 India; 11 Department of Chemistry, College of Science, King Saud University, P.O. Box 2455, 11451 Riyadh, Kingdom of Saudi Arabia; 12 Department of Biology and Chemistry, College of Sciences and Engineering, Southern University, Baton Rouge, LA 70813 USA; 13 Department of Transport Systems, Urgench State University, 220100 Urgench, Uzbekistan

**Keywords:** TCO, Perovskite solar cells, Spray coating, ZnSnO, MgSnO, Optical, Electrical and J–V studies, Chemistry, Energy science and technology, Materials science, Nanoscience and technology

## Abstract

Zinc-doped tin oxide (ZTO) and magnesium-doped tin oxide (MTO) transparent conducting oxide (TCO) thin films were synthesized using the atomizer spray pyrolysis method and annealed at 450 °C for application as front electrodes in perovskite solar cells (PSCs). The precursor molar ratio was varied from 0.1:0.1 to 0.1:0.5 (Zn/Mg:Sn), resulting in film thicknesses ranging from 210 to 513 nm for ZTO and 243 to 688 nm for MTO after annealing. X-ray diffraction analysis confirmed improved crystallinity with increased crystallite size after annealing. Optical studies revealed high transmittance of ~ 76–80% in the visible region. The optical bandgap decreased after annealing, from 3.85 to 3.67 eV for ZTO and from 3.82 to 3.40 eV for MTO. Hall effect measurements confirmed n-type conductivity with enhanced electrical performance at higher molar concentrations. The optimized MTO film exhibited a maximum carrier concentration of 2.78 × 10^20^ cm^−3^, mobility of 31.56 cm^2^/V s, and low resistivity of 1.89 × 10^−4^ Ω cm. Photovoltaic devices fabricated using annealed ZTO and MTO substrates achieved power conversion efficiencies of 3.45% and 6.38%, respectively. The improved device performance of MTO-based PSCs is attributed to its higher conductivity and optical transparency, demonstrating its potential as an alternative cost-effective TCO material for solar cell applications.

**Supplementary Information:**

The online version contains supplementary material available at 10.1038/s41598-026-42690-x.

## Introduction

Transparent Conducting Oxide (TCO) substrates have significant applications in optoelectronic devices, such as solar cells, light-emitting diodes, touch panels and liquid crystal displays^1–5^. Solar cells powered by an inexhaustible source of solar energy are essential for promoting green energy worldwide. This underscores the need to develop new materials for each layer of the solar cells. Indium-doped Tin Oxide (ITO) and Fluorine-doped Tin Oxide (ITO) are the predominant materials in the solar industry, particularly for perovskite solar cells (PSCs) and dye sensitized solar cells (DSSCs), due to their superior optical and electrical characteristics^6,7^. However, the scarcity, high cost and further enhancement of the optical properties of the front electrode are crucial for PSCs, which necessitates alternative solutions^8–10^. Additionally, the two most desirable qualities for transparent conductive films are low sheet resistance and high transparency. However, conductivity and transparency have a trade-off connection, making it challenging to achieve both qualities simultaneously^11^. The ITO substrate had a high transmittance (> 80%) and low sheet resistance (< 40 Ω/sq) at visible wavelengths. However there are several drawbacks that restrict their application in PSCs^11^. Taking these factors into account, substitute TCO materials are required to replace ITO. Our current research focuses on finding new TCO materials and improving the efficiency of PSCs by optimizing one of the primary layers, known as the window layer, through higher solution concentrations, deposition, and annealing temperatures compared with previous studies^12–16^. Numerous materials have been investigated as potential ITO replacements for TCOs, including metal nanowires, hybrid/composites^17^, conducting polymers^18^ and other transition metal oxide materials^19^. Other doped metal oxides (Zn and Mg), such as impurity-doped SnO_2_, have been identified as interesting possibilities in the search for effective ITO replacement materials for PSCs. Zn/Mg/Sn materials are cost-effective with high production rates^16,20–22^. The constructed window layers, utilized in the hole transport layer (HTL) and electron transport layer (ETL), are effective for power generation. In previous studies, we developed new layers and solar cells, demonstrating their impact on efficiency. Zinc Oxide (ZnO) is a semiconductors with high excitation, a wide energy bandgap (3.37 eV), good chemical and thermal stability, binding energy (60 meV) and n-type electrical conductivity. It is abundant, non-toxic and environmentally friend^20–25^. Moreover, ZnO is essential for optoelectronic applications, bioimaging and cancer detection^20^. A number of ways are followed for enhancing the properties of SnO_2_ such as annealing, surfactant coating and addition of impurities^21,22^. To improve the electrical and optical properties of tin oxide materials, magnesium and zinc were chosen as doping materials in this study.

According to Yu et al. the properties of Transparent Conductive Films (TCFs) depend on stoichiometric deviations and impurity types^21^. ZTO, with its high optical transmittance, offers a promising application for TCFs, despite being expensive and less abundant^17^. However, ZTO is challenging to use in flexible electronic devices owing to its low sheet resistance. Prior research has mostly concentrated on the optical and electrical characteristics of multicoating structures. For real-world applications, the flexibility, stability and adherence of multilayer thin films are crucial characteristics^21–23^. Owing to their lower absorption and resistivity values, ZTO and MTO can replace ITO in solar cells^22–24,26–28^.

Analysis of various properties, such as electrical, structural, optical and thickness, suggests the potential of these films for societal and commercial applications.

Mansi Sharma et al. reported that ZnO and MgO-based contacts show higher efficiencies than ITO-based contacts in amorphous silicon thin-film solar cells because of better optical characteristics that facilitate light trapping and higher drift velocity of holes close to the junction^25^. Conducting layers were prepared for Mg and Zn at different molar ratios from 0.1 M:0.1 M to 0.1 M:0.5 M. After annealing, the films exhibited improved conductivity and efficiency owing to the increased charge carriers and enhanced carrier mobility^24,25,29–31^. Magnesium oxide (MgO)-based thin films play an essential role in the photovoltaic industry due to their unique electrical and optical properties^32^. Many coating techniques are available for thin film preparation, including sputtering, spin-coating, and electrodeposition. However, because of its simplicity, flexibility and easy modification of the processing parameters, the atomizer spray pyrolysis (ASP) method can be chosen.

Earlier studies predominantly focus on standalone materials characterization or non-PSC applications, with limited attention to processing window optimization or device-level validation. In contrast, this work presents a comparative and systematic investigation of Zn-doped and Mg-doped tin oxide transparent conducting films synthesized via atomizer spray pyrolysis, emphasizing controlled precursor molarity, annealing-induced phase evolution, and thickness-dependent charge transport. Importantly, the prepared films are directly integrated as transparent electrodes in perovskite solar cells, and their impact on photocurrent generation and power conversion efficiency is experimentally demonstrated. To the best of our knowledge, such a parallel ZTO/MTO study with explicit materials-to-device correlation using spray-pyrolyzed TCOs for PSC applications has not been previously reported.

In this work, CsSnCl_3_ was selected as a lead-free perovskite material to reduce environmental concerns associated with lead-based PSCs. Graphite was employed as a low-cost and stable carbon electrode to enable simple and scalable device fabrication. The primary objective of this study is to evaluate the performance of the newly developed ZTO and MTO transparent conducting oxide substrates rather than to achieve record efficiencies. Therefore, a simplified and stable device architecture was adopted to clearly understand the influence of the TCO layer on photovoltaic performance.

## Experimental

### Preparation of the prime solution

Spray pyrolysis is an inexpensive, flexible, and scalable coating technology used for depositing thin film layers. Process parameters, such as the deposition temperature, carrier gas pressure, solution volume, concentration, molarity ratio and separation between the heated substrate and spray nozzle, were modified during the film coating process^12–16^. The chemicals used were magnesium acetate tetrahydrate [(CH_3_COO)_2_ Mg·4H_2_O], zinc acetate tetrahydrate Zn(CH_3_COO)_2_ 0.2H_2_O,Tin(II) chloride dihydrate (SnCl_2_·2H_2_O) and 2-propanol (CH_3_CHOHCH_3_) with a purity of ≥ 99.0% (Sigma Aldrich). Glass substrates, measuring 30 mm × 30 mm and 8 mm in thickness, were thoroughly cleaned with a soap solution and acetone, followed by ultrasonic bath cleaning using double-distilled water^20,23^. Precursor solutions of zinc acetate, magnesium acetate and tin(II) chloride were dissolved in isopropyl alcohol. To improve dispersion, a few drops of HCl and deionized (DI) water were added while stirring. To maintain the solution stability and maximize the reaction rate, 4–5 drops of ethanolamine were added. The coated film was then annealed, and the properties of both the annealed and unannealed films were analyzed using various characterization techniques. Supplementary Table S1 lists the precursor concentrations used for thin film deposition. The ASP technique provides an economical way to synthesize thin films without the need for high-vacuum systems. This method offers good control over thickness uniformity and adherence to the substrate^33–36^.

### Experimental procedure for the transparent conducting layer preparation

**Fig. 1:**
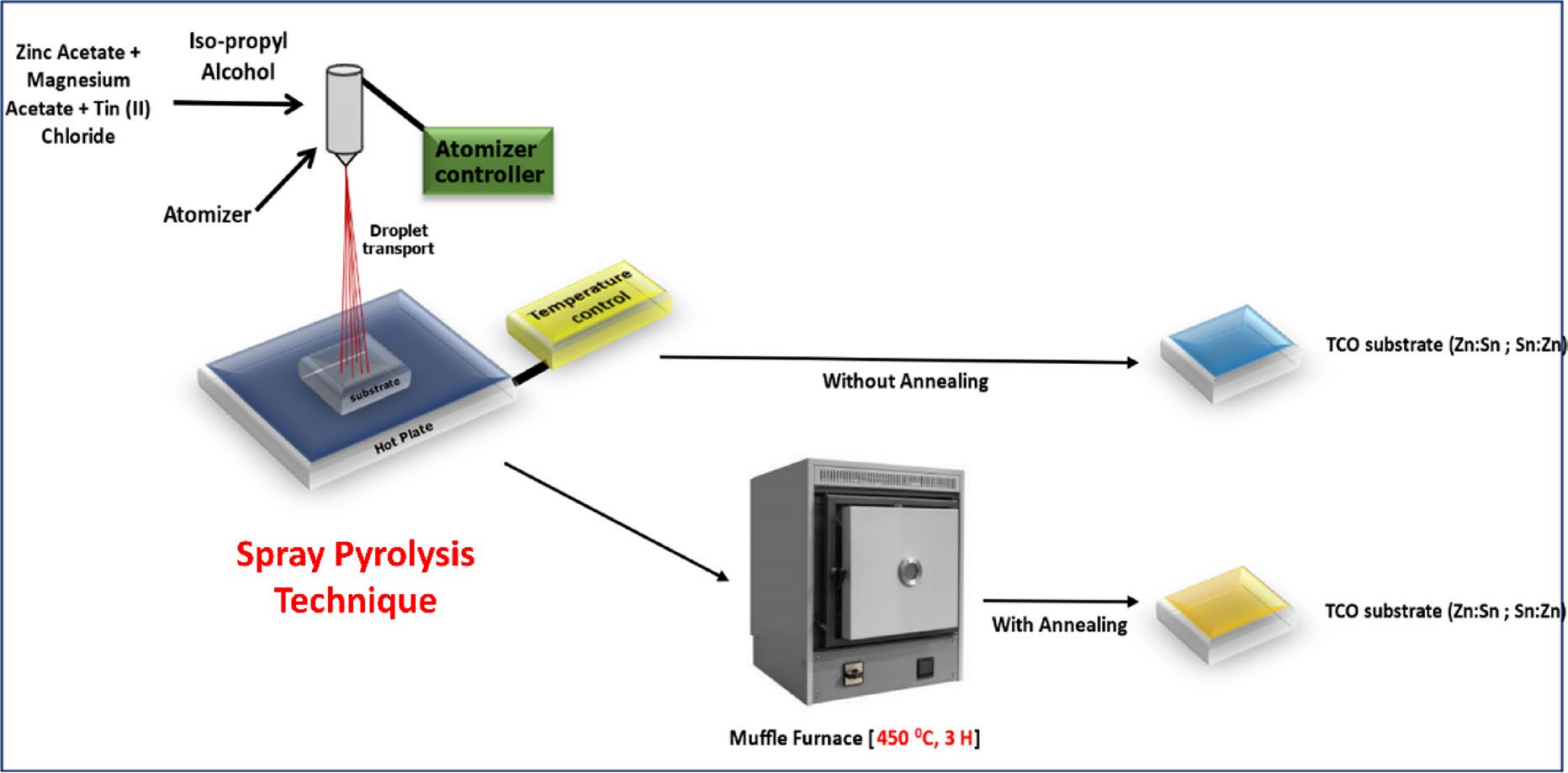
Diagrammatic illustration of the atomiser pyrolysis process used to create thin films.

After preparing the source solution for ZTO and MTO thin films, the following parameters were optimized and kept constant during the manual coating process: the carrier gas pressure was maintained at 0.6 kg/cm^2^, the spray rate for the solution was 30 mL per 15–20 min, and the spray nozzle and substrate were positioned 20 cm apart. The solution volume was fixed at 100 mL (50 mL tin chloride + 50 mL acetate solution). The speed of the spray gun tip in the (x, y) direction was 2 cm every 5 s and the deposition temperature was maintained at 300 ± 10 °C. After reaching the desired temperature, spraying was initiated using a spray gun. The coated film was removed once the substrate reached room temperature and then annealed at 450 ± 10 °C for 2 h. Figure 1 illustrates the experimental technique used for the preparation of the required TCO films.

### Device fabrication of PSCs

#### Electron transport layer

The preparation of the TiO₂ paste followed the procedure outlined by Raguram et al.^37^. To begin, 1 g of TiO₂ powder underwent grinding for 20 min. Next, 0.17 mL of acetic acid and 0.85 mL of deionized (DI) water were combined in a container and mixed thoroughly. This mixture was then added gradually yo the ground TiO₂ powder while blending continued. Finally, Teflon was added as a binder and the mixture was ground using a mortar and pestle until a homogeneous TiO₂ paste was obtained. The prepared TiO₂ paste was then coated onto the prepared TCO substrates using the Doctor Blade technique to form an active layer with a thickness of approximately 0.5 mm. For one hour, the coated substrate was annealed at 450 °C to guarantee the correct adherence and crystallinity of TiO₂ layer’s.

#### Preparation of perovskite layer

A mixture of dimethylformamide (DMF) and dimethyl sulfoxide (DMSO) was used to dissolve cesium chloride (CsCl) and tin (II) chloride (SnCl₂) in a 9:1 volume ratio to create the perovskite precursor solution (CsSnCl₃). Specifically, 0.1 M of CsCl (2.287 g) and 0.1 M of SnCl₂ (3.065 g) were individually dissolved in the solvent mixture and stirred separately for 30 min. The two solutions were then combined and stirred at 70 °C for 1 h using a magnetic stirrer to ensure the complete dissolution of the salts, resulting in a homogeneous precursor solution.

#### Perovskite layer deposition

Nebulizer spray pyrolysis was used to create perovskite films on the surfaces coated with TiO₂/TCO. Before the deposition, the hot plate was cleaned with distilled water and acetone. The substrates were placed on a hot plate (400 °C) under air atmosphere for each deposition. The prepared precursor solution was sprayed onto a heated substrate at a controlled spray rate of 0.75 mL/min using compressed air as the carrier gas. For consistent spraying, an air compressor was attached to a U-shaped glass tube that held the nebulizer. To ensure a consistent coating, the spraying unit was maintained at a fixed distance of 3 cm from the substrate surface. The spraying procedure was repeated until 20 mL of the solution was completely deposited on the substrate. After the deposition, the coated substrates were allowed to cool to room temperature and then annealed in a muffle furnace at 250 °C for 5 min to enhance the crystallinity and adhesion of the perovskite layer.

#### Preparation of hole transport layer

One gram of graphite powder was ground using a mortar and pestle for 20 min. Paraffin oil was then added as a binding agent and the mixture was ground for an additional 30 min to form a uniform graphite paste. Using the doctor blade approach, the formed graphite paste was applied to the substrate, covering a 0.5 mm active area. The coated substrates were subsequently annealed in a muffle furnace at 450 °C for 1 h to ensure the proper adhesion and stability of the hole transport layer.

### Characterisation techniques

After preparing the TCO substrates, various instruments were utilized to analyze their different characteristics, including a Stylus Profilometer, XRD, electrical studies through a Hall Effect measurement system, UV–Vis spectrophotometer, J–V and FESEM analysis. The thickness was measured using a Stylus Profilometer (ZYGO Optical Profilometer) and microgravimetric method. X-ray Diffractometer (PW3071/xx Bracket Diffractometer system XPERT-PRO) was employed to conduct structural analysis. Investigation into conductivity properties, including the determination of material classification (n-type or p-type), mobility, sheet resistance, resistivity and carrier concentration, were carried out using Hall Effect Measurements (Ecopia Instrument). Morphological and compositional analyses were conducted using a field emission scanning electron microscope (FESEM) with an integrated Energy Dispersive X-ray Spectrometer (EDAX). This specific equipment utilized was a ZEISS SIGMA HV model, featuring a Bruker Quantax 200-Z10 as the EDS Detector. To thoroughly examine the optical characteristics, a UV–Vis spectrophotometer (JASCO, V-670) was used. A Keithley Instrument (Tektronix, 2650 Source Meter) was used to measure the J–V characteristics.

## Results and discussion

### Structural analysis

**Fig. 2:**
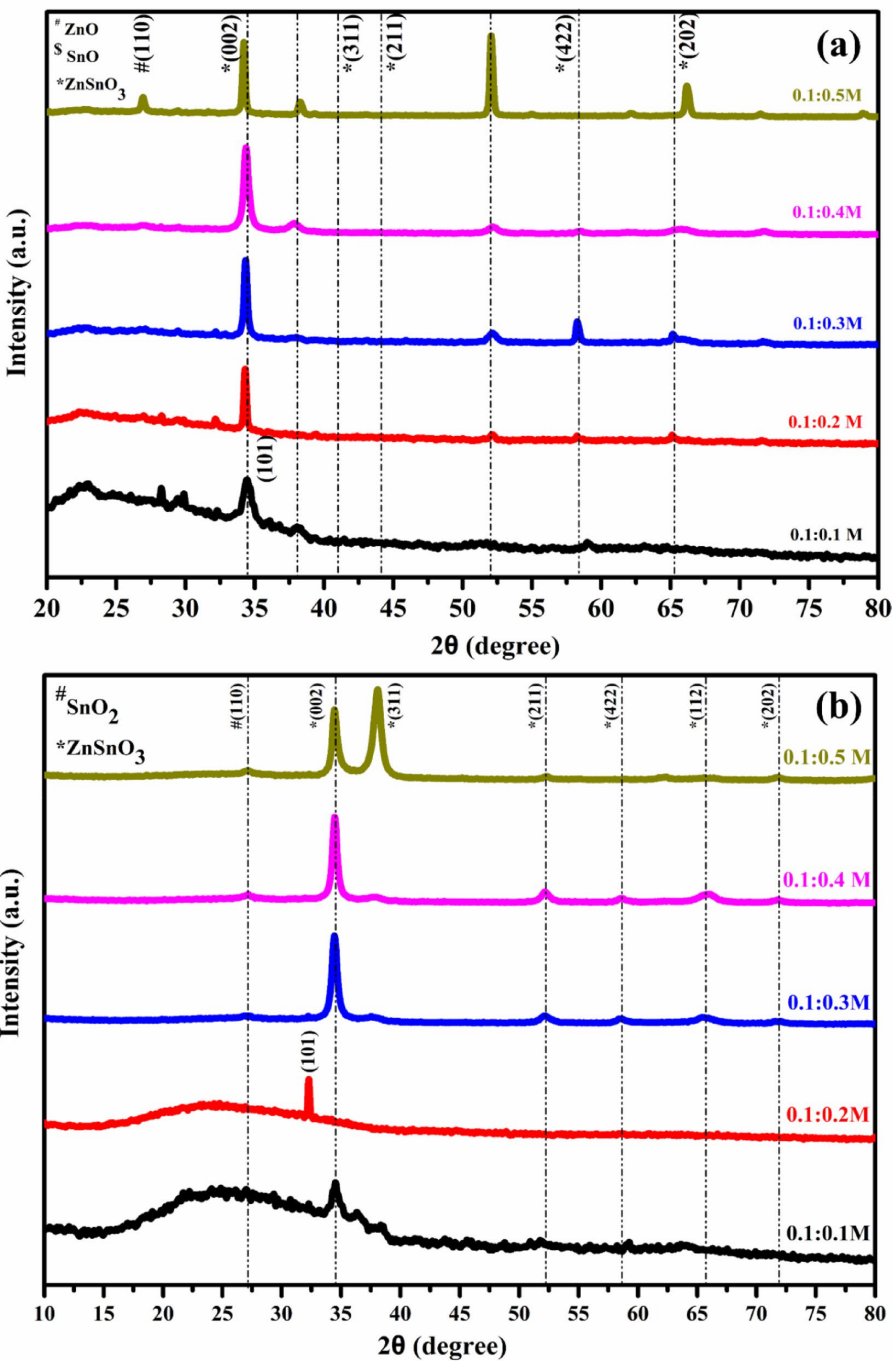

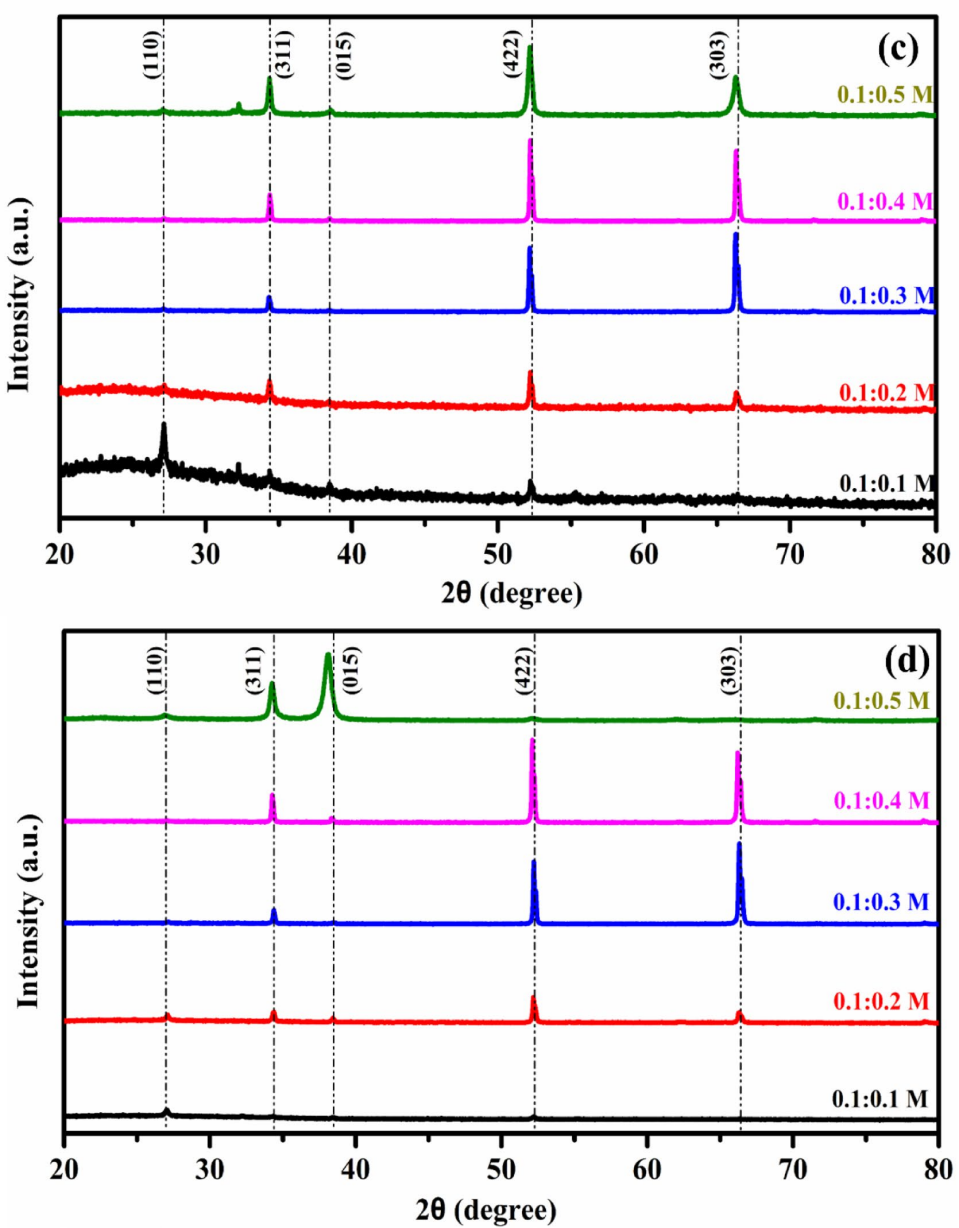
XRD pattern for different molar concentration of (**a**, **b**) ZTO film and (**c**, **d**) MTO film without and with annealing at 450 °C.

The XRD spectra reveal the structural details and crystalline nature of the thin films. XRD patterns are shown in Fig. 2a–d which show both the annealed and non-annealed samples of ZTO and MTO thin films. All the diffraction peaks were indexed to JCPDS and the details are provided in Supplementary Tables S2 and S3. The parameters measured for the structural analysis included the grain size, microstrain, and dislocation density. The hardness and ductility of the material improved after annealing. From Fig. 2a, b, it can be observed that the major peak corresponds to the lattice plane (200), indicating the presence of ZnSnO_3_. This is likely the result of the substitution of zinc ions (Zn^2+^) into the lattice points of the tetragonal Sn nanocrystalline structure. Zn-doped tin oxide exhibited both hexagonal and tetragonal structures. From the XRD patterns, it can be concluded that the increased intensity of the peaks indicates a better crystalline structure as the molar concentration increases. Mixed phases of SnO_2_ and ZnO were obtained without annealing. However, after annealing, these mixed phases were reduced and only the ZnSnO_3_ phase was observed. The structural properties reveal that ZnO nanoparticles have a hexagonal structure, whereas Sn nanoparticles have a tetragonal structure^15^. For the ratios (0.1:0.1 M) and (0.1:0.2 M), the first and second peaks corresponding to 2θ = 28.16° and 2θ = 34.55° are assigned to ZnO. Zn and Sn are oriented in the (110) and preferred orientation along the (002) planes and are crystalline in the hexagonal phase, as confirmed by JCPDS no. (36-1451). The predominant third peak corresponds to the ZnSnO_3_ phase, as confirmed by the JCPDS card no. (11-0274). The second peak corresponding to 2θ = 52.19° is assigned to Sn, oriented in the (211) plane and crystalline in the tetragonal phase, as confirmed by JCPDS no. (041-1445) for films coated with a ratio of 0.1:0.3 M.

For the ratio (0.1:0.4 M), the first and third peaks are at 2θ = 34.47° and a slight peak at 58.60°, corresponding to the preferred orientation along the (002) and (422) planes, respectively with ZnSnO_3_ as the compound. The second peak at 2θ = 52.16° corresponds to the (211) plane, indicating the presence of Sn, as confirmed by JCPDS no. (0410-1451). For the ratio (0.1:0.5), all the peaks at 2θ = 28.89°, 34.41°, 38.08°, 52.30°, and 62.13° correspond to the (002), (222), (211), and (202) planes, indicating ZnO, SnO_2_, and ZnSnO_3_ with JCPDS no. (11-0274). At higher ratios, only the expected ZnSnO_3_ phase is observed. At every ratio, the presence of ZnO indicates the dominance of zinc because of its faster and easier oxidation compared to Sn^16,20^. A JCPDS comparison is provided in Supplementary Tables S4 and S5 for the samples with and without annealing**.**

After annealing, the dominance of ZnO is controlled. For the ratio (0.1:0.2), only one peak is present, corresponding to the ZnO phase oriented on the (101) plane, as confirmed by JCPDS no. (36-1451). For the ratio (0.1:0.3), the first and third peaks are at 2θ = 34.30° and 58.25°, corresponding to the (preferred orientation along the (002) and (422) planes, respectively, with JCPDS no. (11-0274), indicating the cubic structure of ZnSnO_3_. The second peak at 2θ = 52.14° was assigned to Sn, oriented in the (013) plane and crystalline in the tetragonal phase, as confirmed by JCPDS no. (041-1445). For the ratio (0.1:0.4), all the peaks are at 2θ = 34.37°, 37.96°, 52.20°, and 58.47°, corresponding to the (002), (311), (024), and (422) planes, with ZnSnO3 present. For the ratio (0.1:0.5), all the peaks at 2θ = 34.28°, 38.20°, 52.01°, 66.13°, 62.17°, and 71.48° correspond to the (002), (311), (211), (511), (301), and (202) planes, indicating ZnSnO_3_ with JCPDS no. (11-0274). The observed preferential orientation of the (002) plane in ZTO films and the (422) plane in MTO films is attributed to dopant-induced lattice distortion, strain effects, and growth kinetics associated with the atomizer pyrolysis deposition and subsequent annealing. The incorporation of Zn^2^⁺ and Mg^2^⁺ ions into the SnO₂ lattice modifies the surface energy of specific crystallographic planes, thereby promoting non-conventional preferential orientations.

After annealing, the peak intensity varied owing to the lattice parameter variation ensuring an improvement in the crystallinity of the material. The crystallite size increased from 48 to 55 nm, and the microstrain and dislocation density decreased after annealing. This characteristic improves the electrical and optical qualities of the produced ZTO thin films^15,16,20^. The Debye–Scherrer formula is employed to determine the crystalline size, while the following equations are used to compute the microstrain and dislocation density^38^:

1 $$D= \frac{K \lambda }{\beta cos \theta }$$

2 $$\varepsilon =\frac{\beta Cos\theta }{4}$$

3 $$\delta =\frac{1}{{D}^{2}}$$

The XRD spectra of the MTO thin films, both unannealed and annealed at 450 °C, are shown in Fig. 2c, d. All the diffraction peaks were confirmed to correspond to the SnO_2_, MgSnO_3_, and Mg_2_SnO_4_ phases. In the case of (0.1:0.1) ratio, the initial peak observed at 2θ = 27.13° was identified as SnO_2_. This peak exhibited a (110) plane orientation and crystallized in the tetragonal structure, as confirmed by the JCPDS card no. (21-1250). The second peak at the (422) plane is the highest and belongs to the Mg_2_SnO_4_ cubic structure, as confirmed by the JCPDS card no. (73-1625). The presence of Mg_2_SnO_4_ indicates its disproportionation into other phases at higher deposition temperatures^4–8^.

For the ratio (0.1:0.2), the first peak at 2θ = 34.36° corresponds to the (311) plane for Mg_2_SnO_4_ with JCPDS no. (24-0723). The highest peak is at 2θ = 66.35° (303) plane, assigned to MgSnO_3_ with JCPDS no. (30-0798). For the ratio (0.1:0.3), the peaks at 2θ = 34.33°, 52.32°, and 66.45° correspond to the (311), (422), and (303) planes, respectively, and the compounds present are Mg_2_SnO_4_ and MgSnO_3_ with JCPDS no. (24-0723), (73-1625), and (30-0798). For the ratio (0.1 M:0.5 M), the first peak at 2θ = 34.35° corresponds to the (311) plane for Mg_2_SnO_4_ with JCPDS no. (24-0723).

The microstructural characteristics of the material are improved by annealing at a comparatively higher temperature, which also makes the material more compact and uniform^21–24^. The strain and dislocation density decreased when the samples were annealed. A decrease in the dislocation density indicates that the crystallinity of the film increased. Additionally, the materials move into a more ordered phase during annealing, which reduces flaws and shrinkage-related dimensional changes. After annealing, the thickness decreases because of shrinkage^5,6^. The enhanced crystallinity of the prepared films was crucial. A decrease in the thickness after annealing indicates reduced absorption and increased transmittance^21–24^. Supplementary Fig. 1S (a-b) shows the thickness variation of the MTO and ZTO thin films (Table 1).

**Table 1:** Thickness of ZTO and MTO thin films with and without annealing.

	Mol. Conc. of the Precursors Zn/Mg : Sn		Thickness (nm)	
	ZTO		MTO	
	Without annealing	With annealing	Without annealing	With annealing
0.1:0.1	252	210	347	243
0.1:0.2	346	286	426	363
0.1:0.3	417	355	514	459
0.1:0.4	542	428	644	556
0.1:0.5	605	513	789	688

### FTIR spectral analysis

**Fig. 3:**
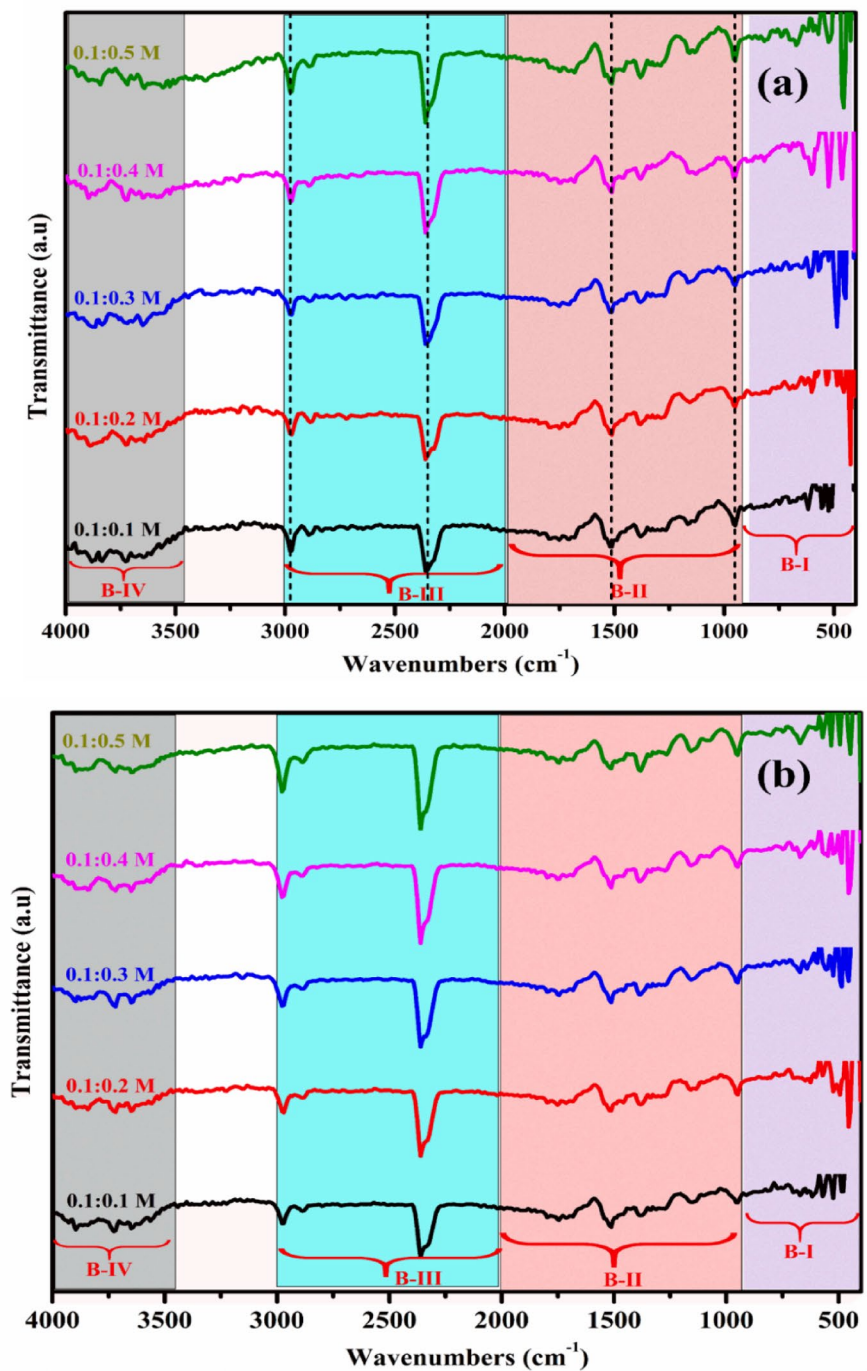

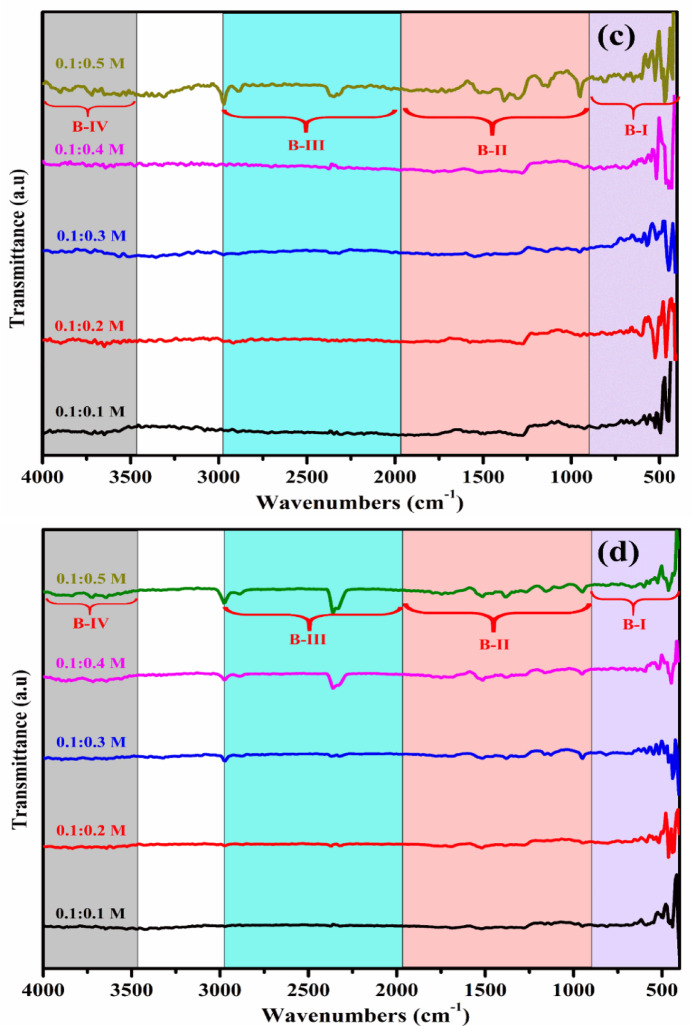
FTIR spectra (**a**, **b**) ZTO film and (**c**, **d**) MTO film without and with annealing at 450 °C.

The functional groups present in the as-prepared and annealed MgSnO₃ and ZnSnO₃ nanocomposites were analyzed using FT-IR spectroscopy. Figure 3a–d show the functional group content of the ZTO and MTO thin films. The materials’ infrared spectra were captured between 4000 and 400 cm^−1^. The molecules present in the unannealed ZnSnO₃ thin films exhibited asymmetric stretching and strong (O–H), (C=C), (C–O) and (C–H). In the FT-IR spectrum, the range of 1000–2000 cm^−1^ shows very weak (C–O), (C–C), and (C–O) bond stretching for all molarities. The peaks between 2000 and 3000 cm^−1^ correspond to the stretching vibrations of C–H and C=C bonds. The presence of hydrogen bonds implicated in O–H bonding is shown by the high peak between 3500 and 4000 cm^−1^^22–25^. The molecules present in the unannealed MgSnO₃ thin films show symmetric stretching, with strong (C–O) and (C=C) bonds, (C–H) and (O–H) bonds. The stretching vibration of Sn–O is observed at 500–600 cm^−1^, while the vibrational stretching of Mg–O–Mg is noted at 400–600 cm^−1^. The vibrational bending of the O–Mg–O bond is observed at around 1400 cm^−1^. O–H stretching and bending were observed at approximately 3500 cm^−1^, indicating water absorption. In the range of 1000 –1500 cm^−1^, there was very weak C–O/C–C stretching for all molarities. Figure 3a, b FTIR spectra for different molar concentrations of ZTO thin films, with and without annealing at 450 °C. The stretching vibrations of the C-H bonds fall between 3000 and 2000 cm^−1^, indicating that each sample absorbed a small quantity of water. The presence of hydrogen bonds implicated in O–H oscillations is indicated by the high peak between 4000 and 3500 cm^−1^. At a substrate temperature of 400 °C, O–H stretching is strong for all molarities of the unannealed MgSnO₃ thin films^23–25,29^. The molecules present in the annealed MgSnO₃ films include alkyl halides (C–I, C–Cl), strong (C–O) and (C=C) bonds, (C–H), and (O–H) bonds, present in various wavenumber ranges of 4000–400 cm^−1^. The O–H stretching and bending vibrations of absorbed water are represented by the broad absorption bands at 3430, 1635, and 1434 cm^−1^. Furthermore, the Mg–O–Mg and O–Sn–O vibrations were represented by two significant absorption bands at 448 and 686 cm^−1^, respectively.

Concurrently, the intensity of O–H vibration diminished, which can be explained by the loss of water molecules through evaporation^25,29,30^. Following the annealing process, both stretching and vibrations were lessened in the MTO and ZTO thin films. Overall, the FTIR spectra exhibit prominent absorption bands in the 400–700 cm^−1^ region, which are attributed to the stretching vibrations of Sn–O, Zn–O, and Mg–O bonds, confirming the formation of the metal oxide network. Peaks observed at higher wavenumbers (around 3200–3500 cm^−1^) correspond to O–H stretching vibrations, indicating the presence of adsorbed moisture or residual hydroxyl groups. After annealing, a noticeable reduction in hydroxyl-related peaks was observed, suggesting improved crystallization and removal of residual organic species. This structural refinement contributes to enhanced electrical conductivity and optical transparency.

### Optical studies

**Fig. 4:**
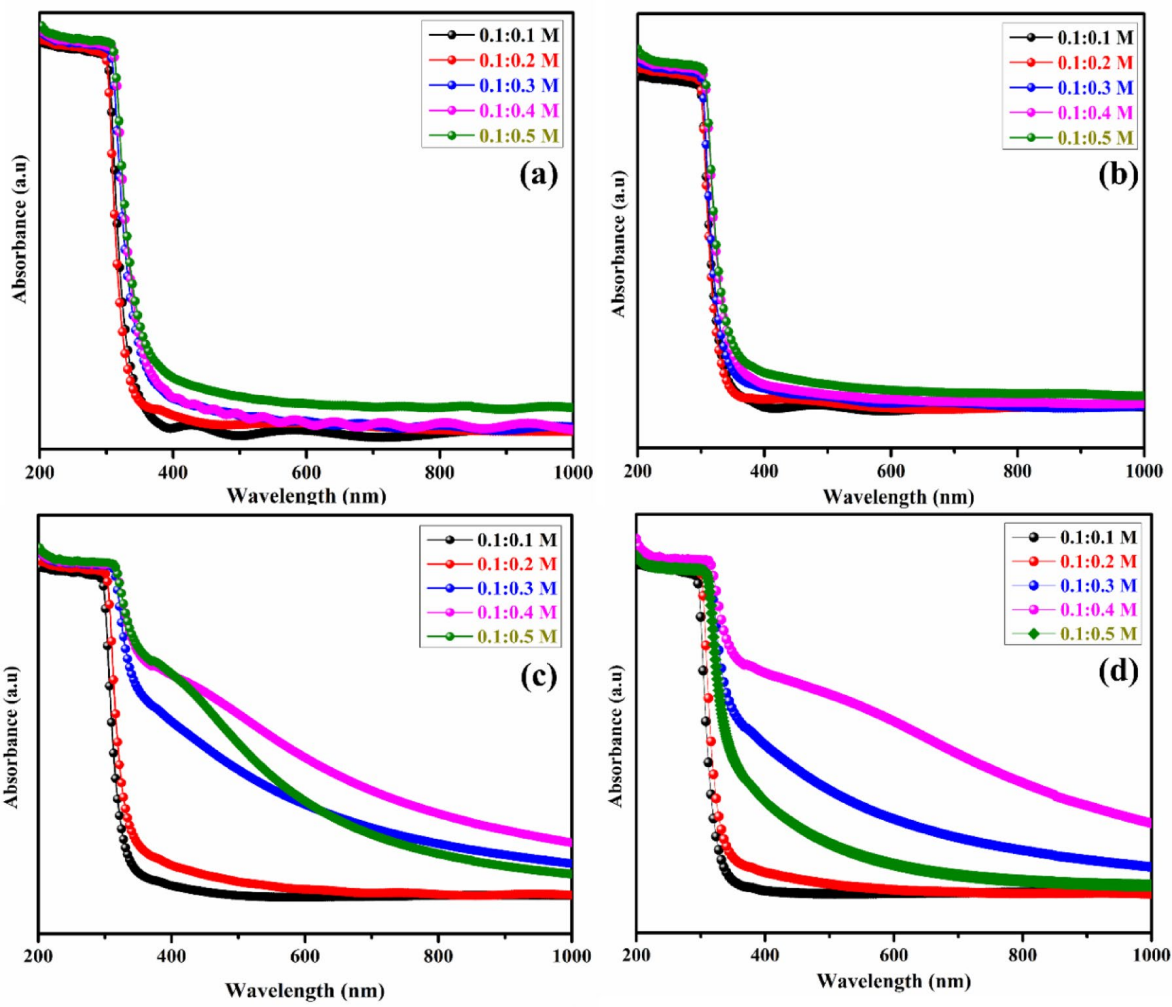
UV–Vis. absorbance spectra analysis of (**a**, **c**) with and without (**b**, **d**) ZTO and MTO thin films.

**Fig. 5:**
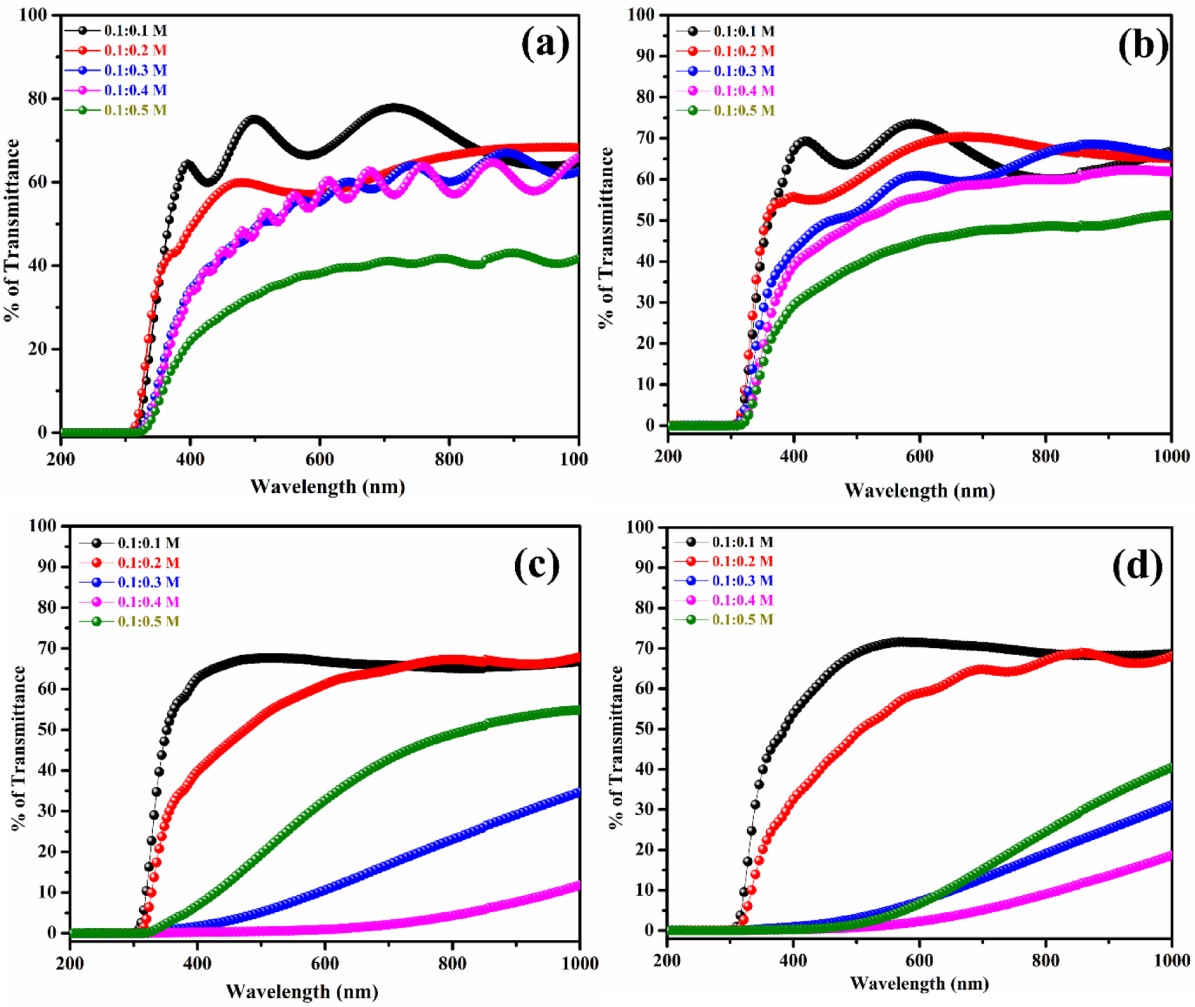
UV–Vis. transmittance spectra analysis of (**a**, **c**) with and without (**b**, **d**) ZTO and MTO thin films.

Absorption and transmission measurements were performed for molar ratios of 0.1:0.1 M, 0.1:0.2 M, 0.1:0. 3 M, 0.1:0.4 M, and 0.1:0.5 M on both substrates at 300 °C. Measurements were performed in the 200–1000 nm range. The transmission spectra of the ZTO and MTO films indicate higher transmission in the visible and IR regions. Figure 4 and 5a–d show the UV–Visible NIR optical studies of the ZTO and MTO thin films before and after annealing.

In the supplementary information, Fig. 2S-5S represent Tauc’s plots for various ZTO and MTO thin films with and without annealing. It was observed that increasing the molar concentration reduced the transmittance range, with the highest transmittance reaching approximately 80% for both the ZTO and MTO thin films. After annealing, the transmittance decreases slightly to approximately 76%. Changes in the material’s local bonding and heterogeneous production of crystalline nuclei are responsible for this reduction^30–32^. The optical band gap values were calculated using Tauc’s plot as indirect allowed transitions which is appropriate for SnO₂-based materials and their doped variants. By altering the carrier concentration, the optical bandgap could be adjusted between 3.67 and 3.86 eV. Table 2 shows that the bandgap decreases as the molar concentration increases for both the unannealed and annealed MgSnO₃ thin films, likely due to the increased tin concentration, which enhances conductivity^25^. The average bandgap for unannealed MTO and ZTO films is 3.60 eV and 3.76 eV, respectively, while the annealed values are 3.49 eV and 3.72 eV, respectively. After annealing at 450 °C for 2 h, a slight reduction in the optical bandgap was observed, which may be attributed to improved crystallinity and reduced structural defects.

**Table 2:** Band gap values of ZTO and MTO thin films with and without annealing.

Mol. Conc. of the precursors	ZTO (eV)		MTO (eV)	
	Without annealing	With annealing	Without annealing	With annealing
0.1:0.1	3.85	3.79	3.82	3.72
0.1:0.2	3.80	3.74	3.71	3.62
0.1:0.3	3.76	3.71	3.56	3.28
0.1:0.4	3.72	3.69	3.48	3.42
0.1:0.5	3.65	3.67	3.45	3.40

Instead, the bandgap narrowing is primarily associated with defect-related localized states, including oxygen vacancies and dopant-induced donor levels, which introduce band tail states near the conduction band edge. Annealing enhances crystallinity while simultaneously activating these shallow defect states, resulting in disorder-induced band tailing (Urbach tails) and a reduced effective optical bandgap. These defect-assisted states also contribute to enhanced carrier transport, explaining the concurrent improvement in electrical conductivity observed in the annealed films^39^. Supplementary Fig. 2S-4S shows the Tauc’s plots for ZTO and MTO thin films. These properties make the films suitable for TCOs owing to their wide bandgaps and high transmittances. Doping may further increase or decrease the optical bandgap by altering the density of localized states^30–33^.

### Field emission scanning electron microscopy (FESEM) analysis

**Fig. 6:**
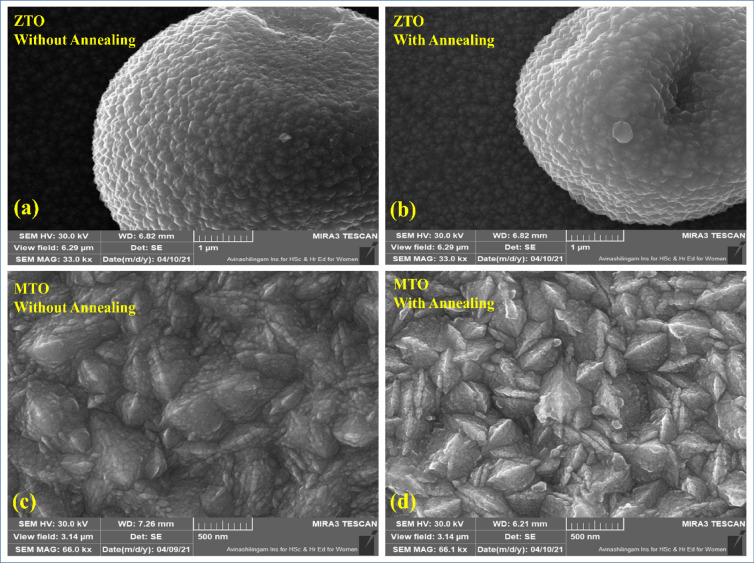
(**a**–**d**) shows that FESEM images of ZTO and MTO films, both with and without annealing.

FESEM is one of the most widely used techniques for characterizing nanomaterials and nanostructures. Figure 6a–d show the FESEM images of the ZTO and MTO films, both with and without annealing. The signals derived from the interactions between the electron and the sample reveal details about the surface morphology of the sample. FESEM results taken at a molarity of 0.5 M for both annealed and unannealed samples. The images reveal that Zn-doped tin oxide nanoparticles generally appear spherical^32–34^, with the particles grouped together to form microspheres^35^. While some regions showed uniform particle size, a non-homogeneous distribution was observed before annealing, and only a few particles displayed non-uniform shapes. The images suggest that Zn doping in metal oxides affects the surface morphology and reduces particle agglomeration as the doping concentration increases. The crystallite size and shape also depend on annealing temperature^32–34^. The microspheres (~ 22 μm) composed of smaller spherical structures (~ 28 nm in diameter) and formed at a deposition temperature of ~ 400 °C showed inhomogeneous sizes (small: 10 μm; large: 18 μm) with irregularly shaped spherical particles^33^. The images also indicate the grain structure growth. The grains are densely packed and forms polyhedral structures. In the case of polyhedral Mg₂SnO₄/SnO₂ nanoparticles, the size decreased to 1 µm–500 nm, while the surface became rougher than that of cubic nanoparticles. The FESEM images of the annealed MgSnO₃ thin films show continuous, tightly bound grains with polyhedral and rhombohedral structures. This may result from more outward surfaces, allowing the release of H₂O and the decomposition of Mg₂SnO₃^34^. The micrographs demonstrate that elevated annealing temperatures resulted in larger grain sizes, consequently enhancing the material’s crystalline structure. The formation of microsphere-like structures in ZTO films may contribute to enhanced light scattering and increased optical path length within the device structure. Additionally, the enlarged grain size after annealing can reduce grain boundary density, potentially minimizing charge carrier recombination and improving charge transport across the TCO/ETL interface. Additionally, the clarity, confinement, and size of the nanoparticles were enhanced after annealing^35,36,40^.

### EDX analysis

**Fig. 7:**
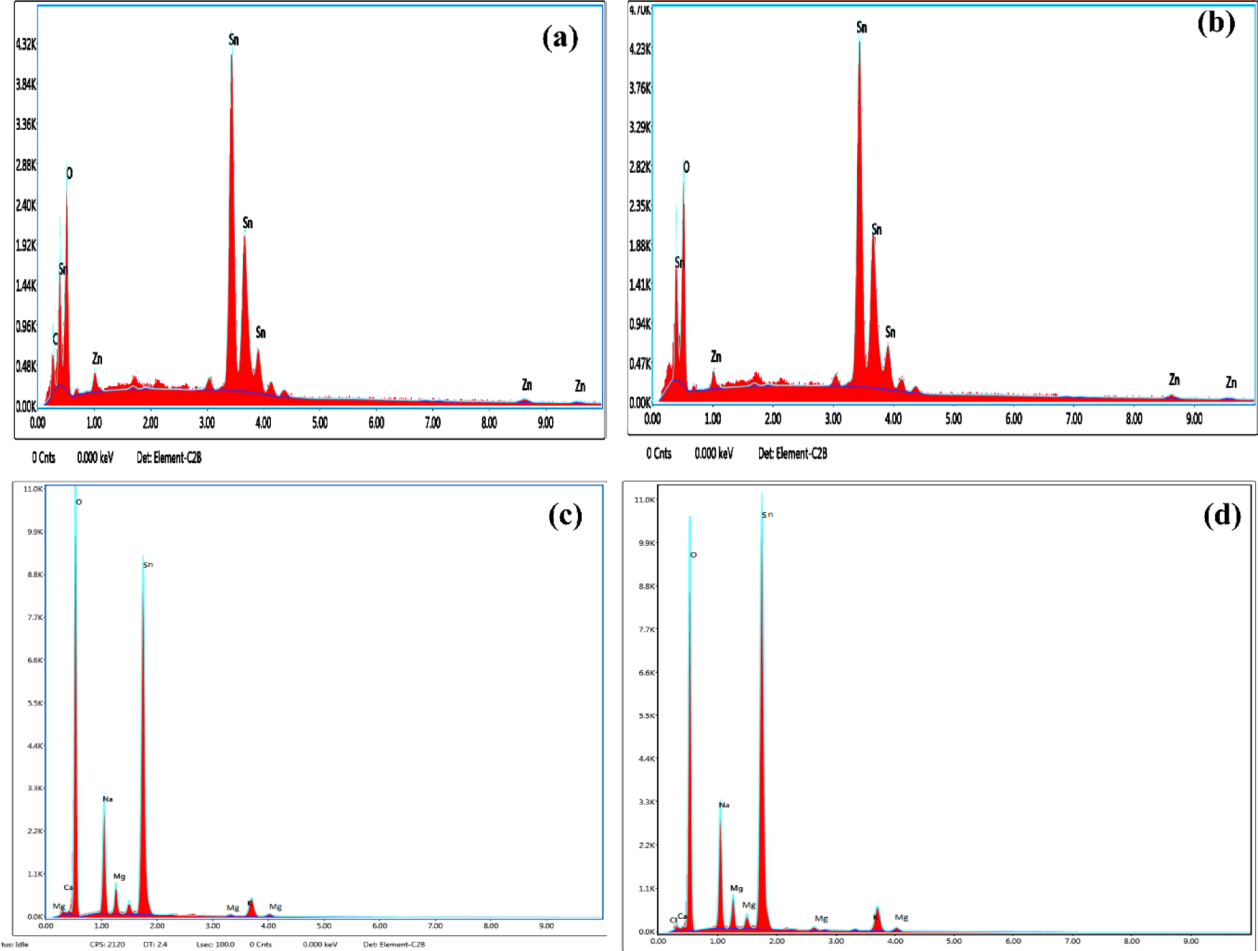
EDS images of (**a**, **b**) ZTO and (**c**, **d**) MTO with and without annealing.

EDX results for ZTO and MTO thin films at a molarity of 0.5 M, both with and without annealing are shown in Fig. 7. Zn, Sn, C and O components were identified in the preparation of zinc acetate and tin(II) chloride solutions. Sn had the highest weight percentage at 81.69%, indicating proper incorporation of Zn atoms into the Zn-doped Sn structure. Zn and O comprise 4.10% and 11.38% of the total weight, respectively. The detected carbon could potentially be attributed to residual chemicals from the solvent used. Figure 5b presents the EDAX results for annealed high molarity substrates, where Sn had an even higher weight percentage of 84.33%, again confirming proper Zn incorporation. A key difference between the unannealed and annealed samples is that unwanted elements such as carbon disappeared after annealing at 450 °C. EDS was used alongside SEM to provide information on the chemical composition of the sample and identify the present elements and their distribution percentages. The EDS analysis of MTO thin films deposited via the nebulizer spray pyrolysis method at 0.5 molarity, shows peaks corresponding to C, O, Mg, and Sn, with the highest peak belonging to Sn. In another EDS analysis of MgSnO₃ thin films, annealed at 450 °C for 3 h, magnesium, tin, chloride, oxygen, and sodium compounds were detected. The sodium signals originate from the glass substrate, which is composed of sodium silicate, and the presence of calcium (Ca) is attributed to the isopropyl alcohol solvent. Magnesium acetate and tin(II) chloride were used as precursors for the detection of magnesium and chloride. The EDX analysis of the Mg-doped SnO₂ nanoparticles examined multiple regions (I and II), showing signals of oxygen, tin, magnesium, carbon, and calcium. These results suggest that the nanoparticles were composed primarily of Sn, O and Mg. EDX spot analysis of different regions of the coated layer revealed that the tin concentration was higher in region I compared to magnesium, with nanoparticles forming clusters.

As the penetration continued, the dopant and matrix concentrations varied slightly, as observed in region II with smaller clusters. However, the magnesium and oxygen levels remained uniform throughout the sample. These findings confirmed that the coated film had a homogeneous composition^36–39^. The EDS results confirm the successful incorporation of Zn and Mg dopants into the SnO₂ matrix, as evidenced by the consistent presence of the respective elemental peaks in both annealed and unannealed samples. The observed variations in conductivity and carrier concentration with increasing precursor molarity suggest effective dopant incorporation into the lattice structure. Although more precise chemical state analysis techniques such as X-ray photoelectron spectroscopy (XPS) would provide deeper insight into oxidation states and bonding configurations, the present EDS analysis confirms the compositional integrity of the prepared films and supports the observed optoelectronic property enhancements (Table 3).

**Table 3:** Weight and Atomic % of the elements (Mg, Sn, Zn, O) present in ZTO and MTO thin films.

Materials prepared	Elements present	Weight (%)	Atomic (%)	Error (%)
ZTO without annealing	C K	2.72	13.37	7.84
	O K	11.48	42.33	8.81
	Sn L	81.69	40.60	2.55
	Zn L	4.10	3.70	19.63
ZTO with annealing	O K	11.42	47.92	8.72
	Sn L	84.33	47.71	2.49
	Zn K	4.25	4.37	19.61
MTO without annealing	Ca K	1.70	6.68	5.56
	Na K	6.23	5.24	8.97
	Sn L	72.45	36.52	2.45
	Mg L	5.67	11.31	7.89
	O L	13.95	40.25	2.34
MTO With annealing	O K	12.34	43.45	4.34
	Sn L	80.54	41.62	3.26
	Mg L	7.12	15.13	8.54

### Hall effect measurements

Thin films with greater molar ratios, such as 0.1:0.3 M, 0.1:0.4 M, and 0.1:0.5 M, are used for Hall Effect investigations. Tables 4 and 5 list the mobility and resistivity values respectively. The negative Hall coefficient confirms the n-type conductivity of the material. Because more oxygen vacancies increase the charge carrier mobility and the grain boundary concentration decreases at lower temperatures, the resistivity decreases^41–46^. The results demonstrate that as the molarity increases, the conductivity also increases while the resistivity decreases, indicating the dual characteristics of transparent conducting oxide (TCO) substrates with high transmittance and conductivity^47^. Thin films with different molar ratios were used for Hall Effect investigations, and the substrate temperature was maintained at 400 °C. The highest mobility was observed for the 0.1:0.5 M ratio, with a carrier concentration of 1.02 × 10^19^ cm^-3^. The negative Hall coefficient confirms the n-type conductivity of the material. The conductivity strongly depends on the composition, with an increased tin concentration leading to enhanced conductivity. The substitution of Zn^2^⁺ and Mg^2^⁺ ions into the SnO₂ lattice can induce lattice distortion and modify donor density, thereby influencing the band structure and enhancing carrier transport. The increase in carrier concentration and mobility with precursor molarity suggests effective dopant incorporation and improved electrical performance. The optimized films exhibited a resistivity of 2.8 × 10^−4^ Ω cm, high optical transmittance of 82% at 550 nm, carrier concentration of 1.6 × 10^20^ cm^−3^, and mobility of 14.2 cm^2^/V s. These parameters confirm the suitability of the prepared films as TCO layers for PSC applications.

**Table 4:** Measurements of hall parameters of ZTO thin films with and without annealing.

Mol. Conc. of the Precursors	Without Annealing	With Annealing	Without Annealing	With Annealing		Without Annealing	With Annealing
	Mobility (cm^2^)/Vs		Resistivity (ρ) (Ω cm)			Carrier concentration (cm^−3^)	
0.1:0.3	15.85	27.80	8.01 × 10^−2^	7.39 × 10^−3^		5.11 × 10^17^	3.41 × 10^18^
0.1:0.4	17.84	30.19	6.72 × 10^−3^	3.06 × 10^−3^		3.46 × 10^18^	2.11 × 10^19^
0.1:0.5	19.43	33.40	2.74 × 10^−4^	2.37 × 10^−4^		2.45 × 10^19^	1.02 × 10^19^

**Table 5:** Measurements of hall parameters of MTO thin films with and without annealing.

Mol. Conc. of the Precursors	Without Annealing	With Annealing	Without Annealing		With Annealing	Without Annealing	With Annealing
	Mobility (cm^2^)/Vs		Resistivity (ρ) (Ω cm)			Carrier concentration (cm^-3^)	
0.1:0.3	17.95	28.72	6.85 × 10^−2^	5.38 × 10^−3^		7.87 × 10^18^	6.67 × 10^18^
0.1:0.4	18.58	29.56	4.10 × 10^−3^	3.08 × 10^−3^		5.23 × 10^18^	4.29 × 10^19^
0.1:0.5	19.89	31.56	2.56 × 10^−3^	1.89 × 10^−4^		3.47 × 10^19^	2.78 × 10^20^

### J–V characteristics

**Fig. 8:**
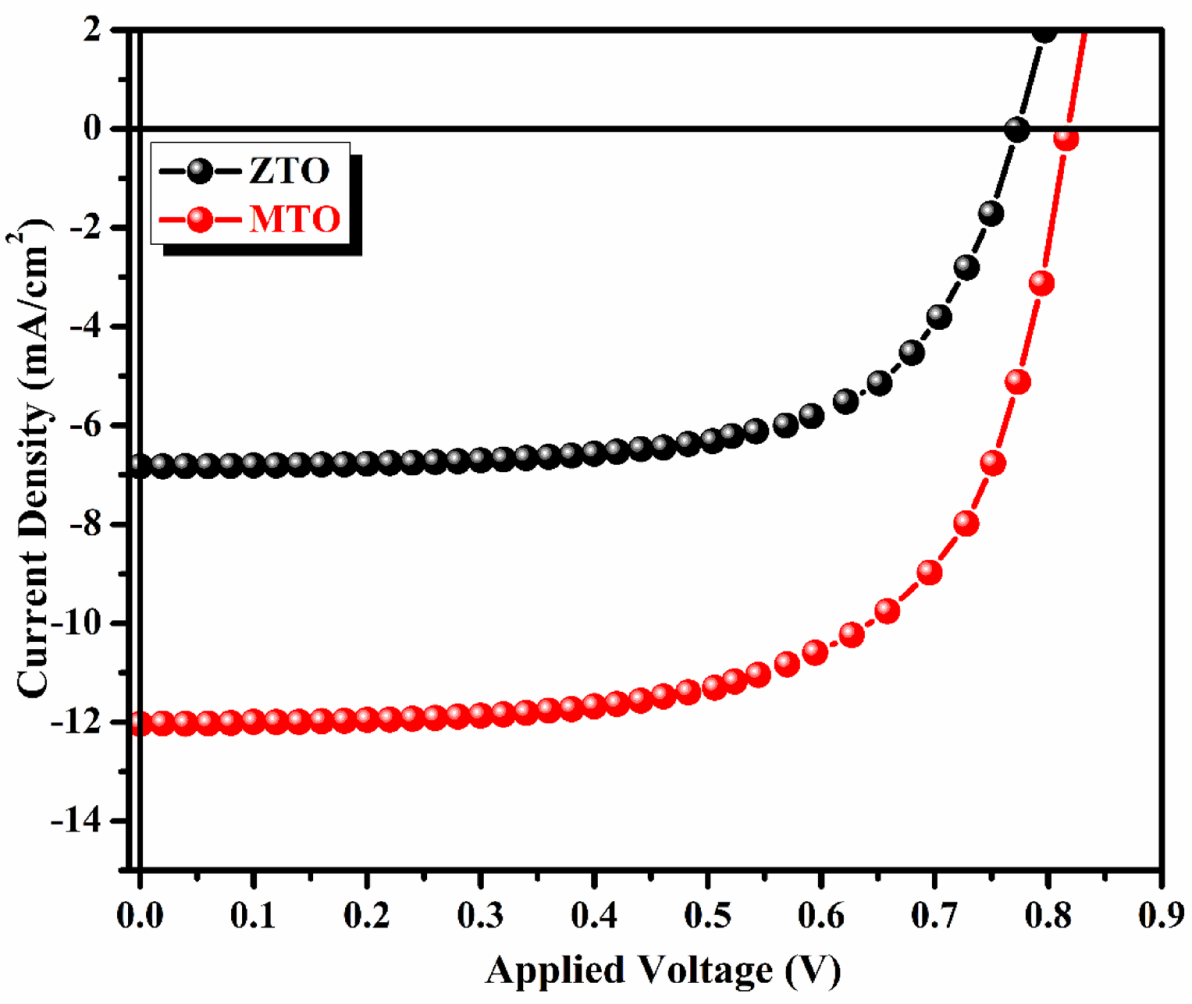
J–V characteristics ZTO and MTO based PSCs for 0.1: 0.5 M conc.

A current–voltage (J–V) measurement was used to calculate the PCE of the solar cell. A solar simulator was employed to replicate 1 sun light intensity (100 mW/cm^2^) for measuring the photocurrent and voltage of the perovskite solar cells using a source meter. The device’s active region measured about 0.25 cm^2^. The current density voltage (J–V) characteristics of perovskite solar cells utilizing ZTO and MTO as transparent conductive oxide (TCO) substrates are depicted in Fig. 8.

The solar cells parameters of the ZTO and MTO based PSCs are listed in the Table 6. The characteristic parameters were extracted from the curves using the following relation;

4 $$FF = \frac{{J_{max} \times V_{max} }}{{J_{SC} \times V_{OC} }}$$

5 $$PCE = \frac{{P_{max} }}{{P_{in} }} = FF\frac{{V_{OC} J_{SC} }}{{P_{in} }}$$

**Table 6:** Solar cells parameters of ZTO and MTO based perovskite solar cells.

Samples	Voc (V)	Jsc (mA/cm^2^)	FF	Η (%)
ZTO	0.77	6.9	0.64	3.45
MTO	0.82	12.04	0.65	6.38

Where, ${P}_{in}$ is the incident power (100 mW/cm^2^). The performance of perovskite solar cells (PSCs) based on ZTO and MTO thin films was evaluated using J–V measurements. The open-circuit voltage (*V*_*oc*_) of ZTO and MTO was determined to be 0.75 V and 0.79 V, respectively. The higher *V*_*oc*_ observed for MTO can be attributed to its lower resistivity and higher conductivity, as supported by Hall effect studies. Hall coefficient measurements confirmed the n-type conductivity of the films, and the highest carrier concentration (2.78 × 10^20^ cm^−3^) was observed for the 0.1:0.5 M molar ratio. This indicates that increasing the molarity enhances the conductivity by reducing the resistivity, which is a result of the increased tin concentration and oxygen vacancies, facilitating improved charge carrier mobility. The higher transmittance percentage of MTO further reinforces its superior performance compared that with of ZTO. MTO’s enhanced transparency allows more incident light to pass through the TCO layer and reach the perovskite layer, leading to increased photon absorption and efficient electron extraction. This improvement is reflected in the short-circuit current density (Jsc), which was recorded at 6.91 mA/cm^2^ for ZTO and 12.04 mA/cm^2^ for MTO. These enhancements in *V*_*oc*_ and *J*_*sc*_ directly contribute to the power conversion efficiencies (PCEs) of the devices. The PCEs were calculated as 3.45% for ZTO-based PSCs and 6.38% for MTO-based PSCs. The combined effects of high transmittance and conductivity, as demonstrated by Hall effect and transmittance studies, underscore the dual functionality of MTO as a transparent conducting oxide (TCO) with significant potential for improving the efficiency of PSCs. The fabricated PSCs in this study are intended as proof-of-concept devices to evaluate the practical integration of the developed ZTO and MTO transparent conducting oxide substrates. The primary aim is to assess the influence of TCO properties on device performance rather than to achieve state-of-the-art efficiencies. The moderate efficiencies obtained may be limited by interfacial recombination at the TCO/TiO₂ interface, series resistance effects, and possible energy level mismatch between the layers. Since both devices were fabricated under identical conditions, the observed performance variation primarily reflects the difference in electrical conductivity and optical transparency of the TCO substrates. Future studies will include benchmarking against standard FTO/ITO electrodes to further validate performance improvements.

## Conclusion

In this investigation, non-identical TCO materials were prepared based on ZnSn and MgSn combinations. This study focused on optimizing the solution concentration and annealing temperature. Both films were analyzed before and after annealing, and in both cases, the crystallite size increased after annealing. Hall effect measurements revealed that the ZTO and MTO films exhibited n-type conductivity due to high electron concentration, with conductivity increasing as the molarity increased. Both films demonstrated high transmittance and low resistivity on the order of 10^−4^ Ω cm for higher tin-doped substrates, indicating their suitability as window layers in perovskite solar cells (PSCs). EDAX confirmed the presence of the required elements, while FESEM analysis revealed microsphere formation and grain growth after annealing. Among the two materials, MgSnO films exhibited superior photovoltaic performance due to their enhanced conductivity and optical transparency. Although the results demonstrate promising potential for photovoltaic applications, further investigations on long-term stability, environmental durability, and large-area scalability are necessary before considering practical or industrial implementation.

## Supplementary Information

Below is the link to the electronic supplementary material.

Supplementary Material 1

## Data Availability

The datasets generated and/or analyzed during the current study are available from the corresponding author on reasonable request.
