# Supplementary material for: Development of Zn1−xSnxO and Mg1−xSnxO transparent conducting oxide thin films for perovskite solar cell applications
Source: Sci Rep. 2026 Apr 3;16:15968. doi: 10.1038/s41598-026-42690-x (PMC13194867; doi:10.1038/s41598-026-42690-x)
Supplement: Supplementary file 1 — Supplementary Material 1 [file 41598_2026_42690_MOESM1_ESM.docx]

**Supplementary Materials**

**Table S1.** Concentration of the precursor used for film deposition

| **Molarity concentration of the precursors**  **Zn:Sn; Mg:Sn (M)** | **Weight of**  **Zinc Acetate**  **(g)** | **Weight of Magnesium acetate**  **(g)** | **Weight of Tin**  **Chloride (g)** | **Volume of**  **Iso**  **Propyl**  **Alcohol (ml)** |
| --- | --- | --- | --- | --- |
| 0.1:0.1 | 1.42 | 1.39 | 1.46 | 100 |
| 0.1:0.2 |  |  | 3.02 |  |
| 0.1:0.3 |  |  | 4.40 |  |
| 0.1:0.4 |  |  | 5.85 |  |
| 0.1:0.5 |  |  | 7.29 |  |

**Table S2.** JCPDS comparison of ZTO thin films without annealing

| **Molarity concentration of the precursors**  **Zn:Sn; Mg:Sn (M)** | **2θ (deg)** | **d (Å)** | **(hkl)** | **JCPDS card no  & phase** |
| --- | --- | --- | --- | --- |
| **0.1:0.1** | 28.16 | 3.2101 | (110) | ZnO (36-1451) |
|  | 31.55 | 2.4737 | (101) | ZnO (36-1451) |
|  | 34.30 | 2.6014 | (200) | ZnSnO_3_ (11-0274) |
| **0.1:0.2** | 32.28 | 2.5703 | (101) | ZnO (36-1451) |
|  | 34.32 | 2.6325 | (200) | ZnSnO_3_ (11-0274) |
| **0.1:0.3** | 34.42 | 2.6052 | (200) | ZnSnO_3_ (11-0274) |
|  | 52.19 | 1.7526 | (211) | SnO2 (041-1445) |
|  | 58.53 | 1.5769 | (422) | ZnSnO_3_ (11-0274) |
| **0.1:0.4** | 34.47 | 2.6015 | (200) | ZnSnO_3_ (11-0274) |
|  | 37.91 | 2.3852 | (222) | ZnSnO_3_ (11-0274) |
|  | 52.16 | 1.7535 | (211) | SnO2 (0410-1451) |
|  | 58.60 | 1.5753 | (422) | ZnSnO_3_ (11-0274) |
| **0.1:0.5** | 34.41 | 2.6057 | (200) | ZnSnO_3_ (11-0274) |
|  | 38.08 | 2.3628 | (222) | ZnSnO_3_ (11-0274) |
|  | 52.30 | 1.7489 | (211) | SnO2 (0410-1451) |
|  | 62.13 | 1.4935 | (202) | ZnSnO_3_ (11-0274) |

**Table S3.** JCPDS comparison of ZTO thin films with annealing

| **Molarity concentration of the precursors**  **Zn:Sn; Mg:Sn (M))** | **2θ (deg)** | **d(Å)** | **hkl** | **JCPDS card no**  **& Phase** |
| --- | --- | --- | --- | --- |
| **0.1:0.1** | 34.62 | 2.60394 | 200 | ZnSnO_3_ (11-0274) |
| **0.1:0.2** | 32.21 | 2.50109 | 101 | ZnO (36-1451) |
| **0.1:0.3** | 34.30 | 2.61395 | 200 | ZnSnO_3_ (11-0274) |
|  | 54.14 | 1.75418 | 024 | ZnSnO_3_ (11-0274) |
|  | 58.25 | 1.58394 | 422 | ZnSnO_3_ (11-0274) |
|  | 65.38 | 1.42729 | 112 | Sn$O_{2}$(01-0657) |
| **0.1:0.4** | 34.37 | 2.60877 | 200 | ZnSnO_3_ (11-0274) |
|  | 37.96 | 2.37628 | 311 | ZnSnO_3_ (11-0274) |
|  | 54.20 | 1.75225 | 024 | ZnSnO_3_ (11-0274) |
|  | 58.47 | 1.57829 | 422 | ZnSnO_3_ (11-0274) |
| **0.1:0.5** | 26.93 | 3.31053 | 110 | Sn$O_{2}$(041-1445) |
|  | 34.28 | 2.62151 | 200 | ZnSnO_3_ (11-0274) |
|  | 38.20 | 2.35564 | 311 | ZnSnO_3_ (11-0274) |
|  | 54.01 | 1.75805 | 024 | ZnSnO_3_ (11-0274) |
|  | 62.17 | 1.49305 | 511 | ZnSnO_3_ (11-0274) |
|  | 66.13 | 1.41301 | 301 | ZnSnO_3_ (11-0274) |
|  | 71.48 | 1.31976 | 202 | ZnSnO_3_ (11-0274) |

**Table S4.** JCPDS comparison of MTO thin films without annealing

| **Molarity concentration of the precursors**  **Zn:Sn; Mg:Sn (M)** | **2θ (deg)** | **d (Å)** | **hkl** | **JCPDS Card No  &  Phase** |
| --- | --- | --- | --- | --- |
| **0.1:0.1 M** | 27.0342 | 3.29834 | 110 | (21-1250) SnO_2_ |
|  | 52.2054 | 1.75220 | 422 | (73-1625) Mg_2_SnO_4_ |
| **0.1:0.2 M** | 27.0896 | 3.29171 | 110 | (21-1250) SnO_2_ |
|  | 34.4003 | 2.60707 | 311 | (24-0723) Mg_2_SnO_4_ |
|  | 38.4068 | 2.34382 | 015 | (30-0798) MgSnO_3_ |
|  | 52.1837 | 1.75288 | 422 | (73-1625) Mg_2_SnO_4_ |
|  | 66.2601 | 1.41057 | 303 | (30-0798) MgSnO_3_ |
| **0.1:0.3 M** | 34.3733 | 2.60906 | 311 | (24-0723) Mg_2_SnO_4_ |
|  | 38.4749 | 2.33983 | 015 | (30-0798) MgSnO_3_ |
|  | 52.2147 | 1.75046 | 422 | (73-1625) Mg_2_SnO_4_ |
|  | 52.3655 | 1.75011 | 422 | (73-1625) Mg_2_SnO_4_ |
|  | 66.3086 | 1.40849 | 303 | (30-0798) MgSnO_3_ |
|  | 66.5023 | 1.40835 | 303 | (30-0798) MgSnO_3_ |
| **0.1:0.4 M** | 34.2697 | 2.61670 | 311 | (24-0723) Mg_2_SnO_4_ |
|  | 38.3430 | 2.34758 | 015 | (30-0798) MgSnO_3_ |
|  | 52.2604 | 1.75339 | 422 | (73-1625) Mg_2_SnO_4_ |
|  | 66.2051 | 1.41044 | 303 | (30-0798) MgSnO_3_ |
|  | 66.3892 | 1.41047 | 303 | (30-0798) MgSnO_3_ |
| **0.1:0.5 M** | 26.9617 | 3.30704 | 110 | (21-1250) SnO_2_ |
|  | 34.2765 | 2.61620 | 311 | (24-0723) Mg_2_SnO_4_ |
|  | 38.0993 | 2.36203 | 015 | (30-0798) MgSnO_3_ |
|  | 52.1052 | 1.75534 | 422 | (73-1625) Mg_2_SnO_4_ |
|  | 62.0297 | 1.49621 | 303 | (30-0798) MgSnO_3_ |

**Table S5.** JCPDS comparison of MTO thin films with annealing

| **Molarity concentration of the precursors**  **Zn:Sn; Mg:Sn (M)** | **2θ (deg)** | **d  (Å)** | **hkl** | **JCPDS Card No & Phase** |
| --- | --- | --- | --- | --- |
| **0.1:0.1 M** | 27.1312 | 3.28676 | 110 | (21-1250) SnO_2_ |
|  | 52.2666 | 1.75030 | 422 | (73-1625) Mg_2_SnO_4_ |
| **0.1:0.2 M** | 34.3673 | 2.60950 | 311 | (24-0723) Mg_2_SnO_4_ |
|  | 52.2189 | 1.75178 | 422 | (73-1625) Mg_2_SnO_4_ |
|  | 66.3544 | 1.40880 | 303 | (30-0798) MgSnO_3_ |
| **0.1:0.3 M** | 34.3323 | 2.61208 | 311 | (24-0723) Mg_2_SnO_4_ |
|  | 52.1730 | 1.75177 | 422 | (73-1625) Mg_2_SnO_4_ |
|  | 52.3206 | 1.75151 | 422 | (73-1625) Mg_2_SnO_4_ |
|  | 66.2685 | 1.40925 | 303 | (30-0798) MgSnO_3_ |
|  | 66.4584 | 1.40918 | 303 | (30-0798) MgSnO_3_ |
| **0.1:0.4 M** | 34.3814 | 2.60846 | 311 | (24-0723) Mg_2_SnO_4_ |
|  | 52.2134 | 1.75050 | 422 | (73-1625) Mg_2_SnO_4_ |
|  | 66.3027 | 1.40861 | 422 | (73-1625) Mg_2_SnO_4_ |
|  | 66.3027 | 1.40860 | 303 | (30-0798) MgSnO_3_ |

**
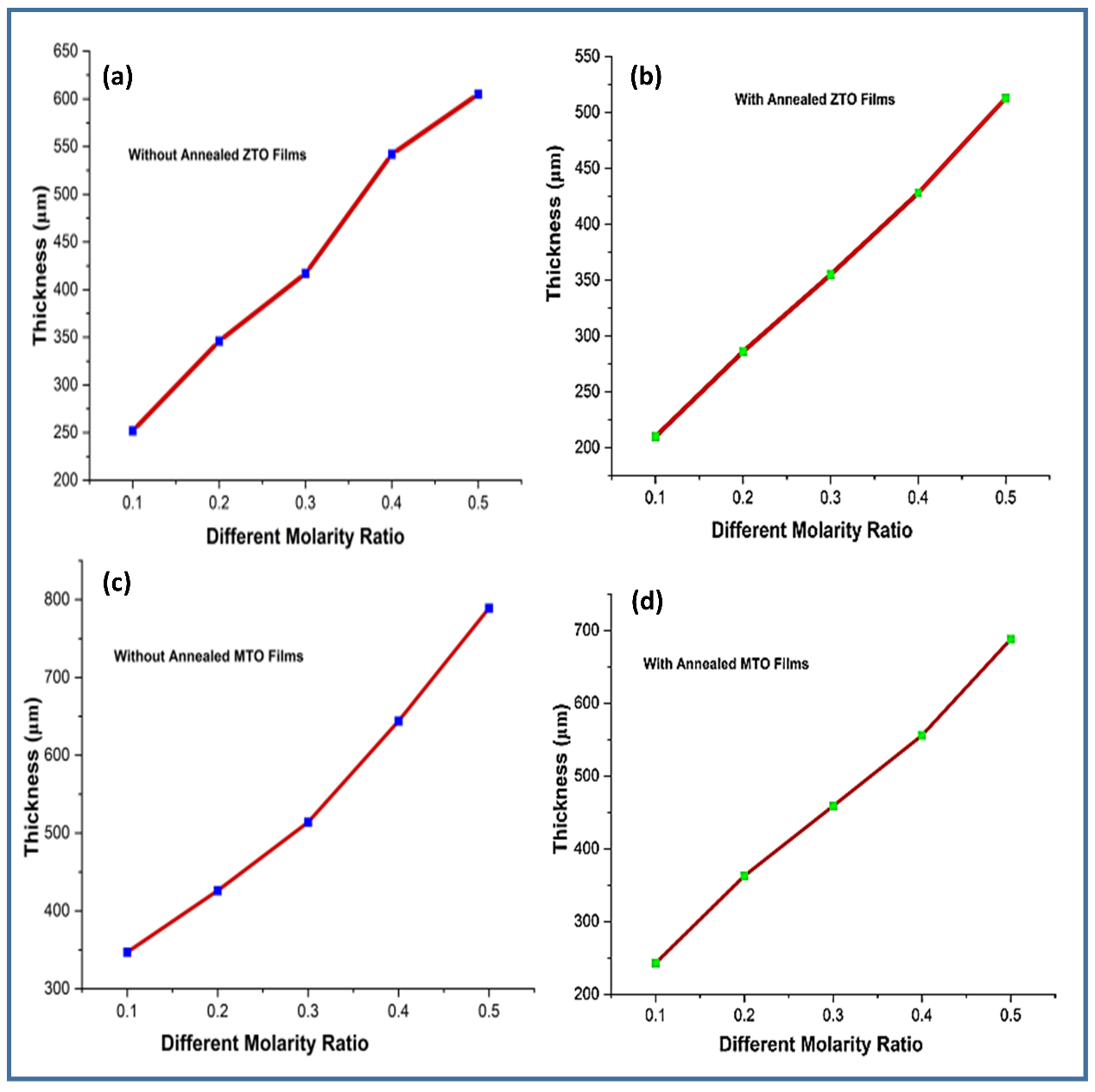
**

**Fig. 1S (a-b)** Thickness variation of ZTO film with and without annealed and **(c-d)** Thickness variation of MTO film with and without annealed


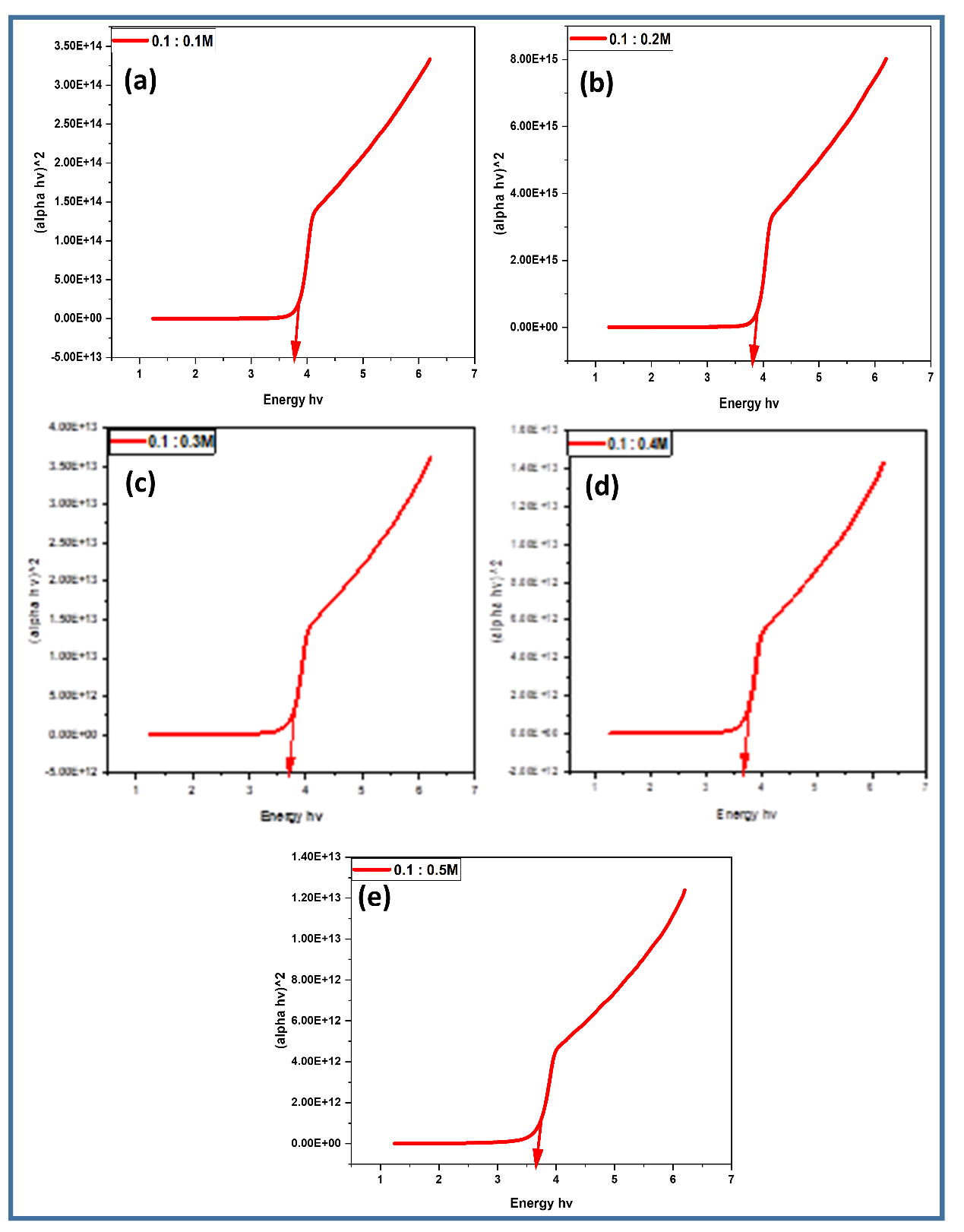


**Fig. 2S (a-e)** Tauc’s plot for ZTO thin films for different molar concentration without annealed


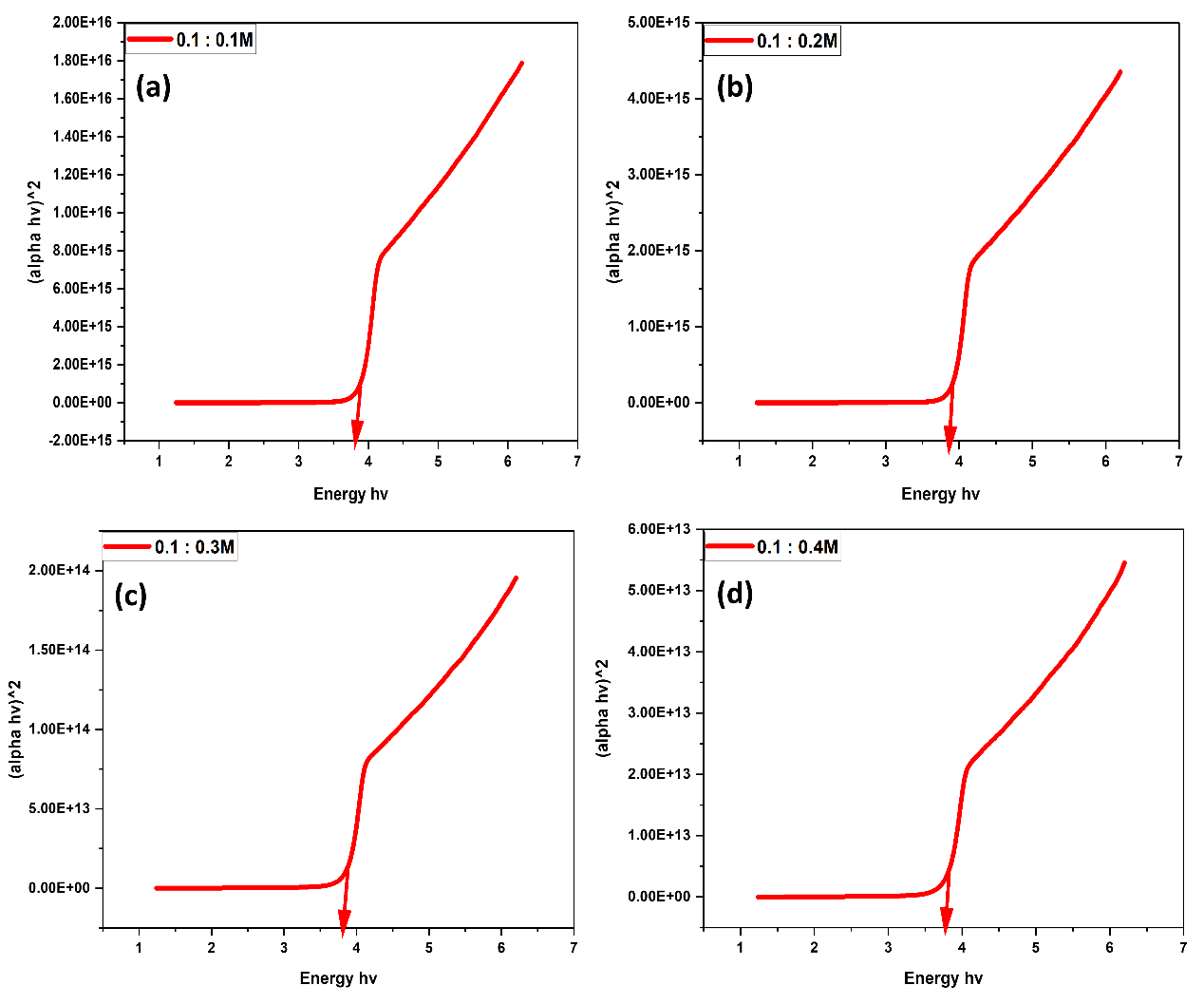
**Fig. 3S (a-e)** Tauc’s plot for ZTO thin films for different molar concentration with annealed

**
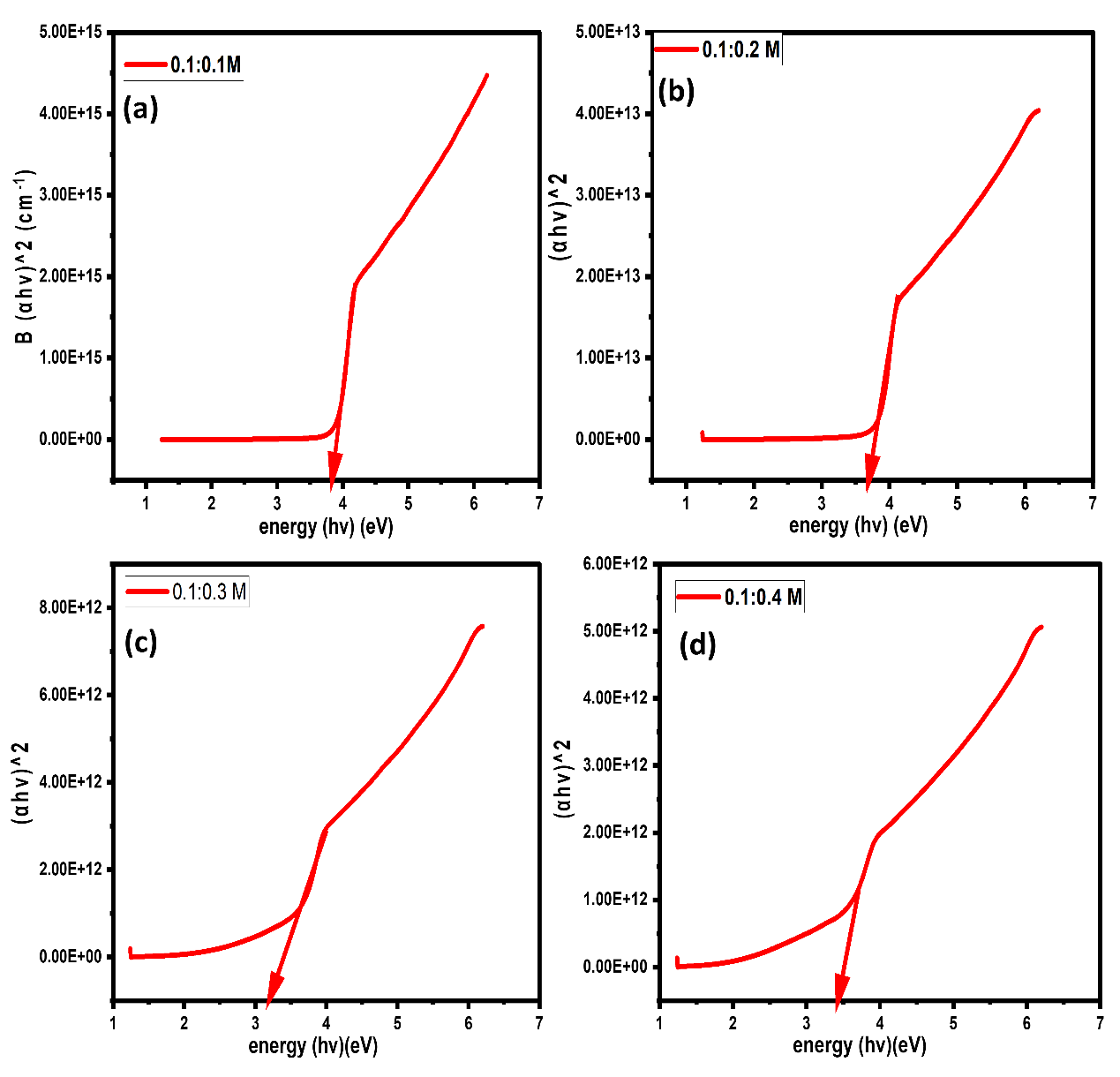
**

**Fig. 4S** (a-d) Tauc’s plot for MgSn$O_{3}$thin films for different molar concentration without annealed

**
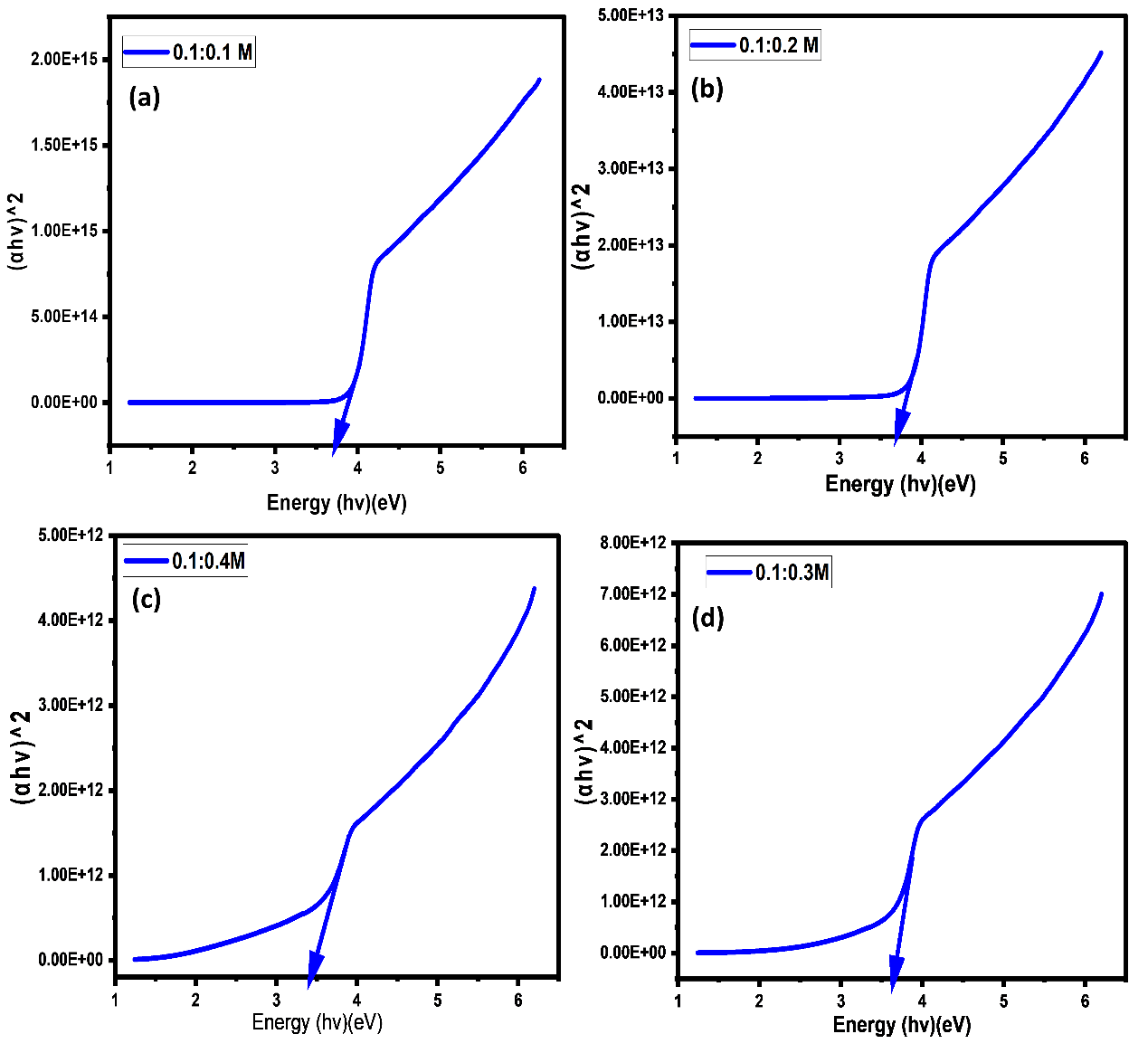
**

**Fig. 5S (a-d)** Tauc’s plot for MgSn$O_{3}$thin films for different molar concentration with annealed


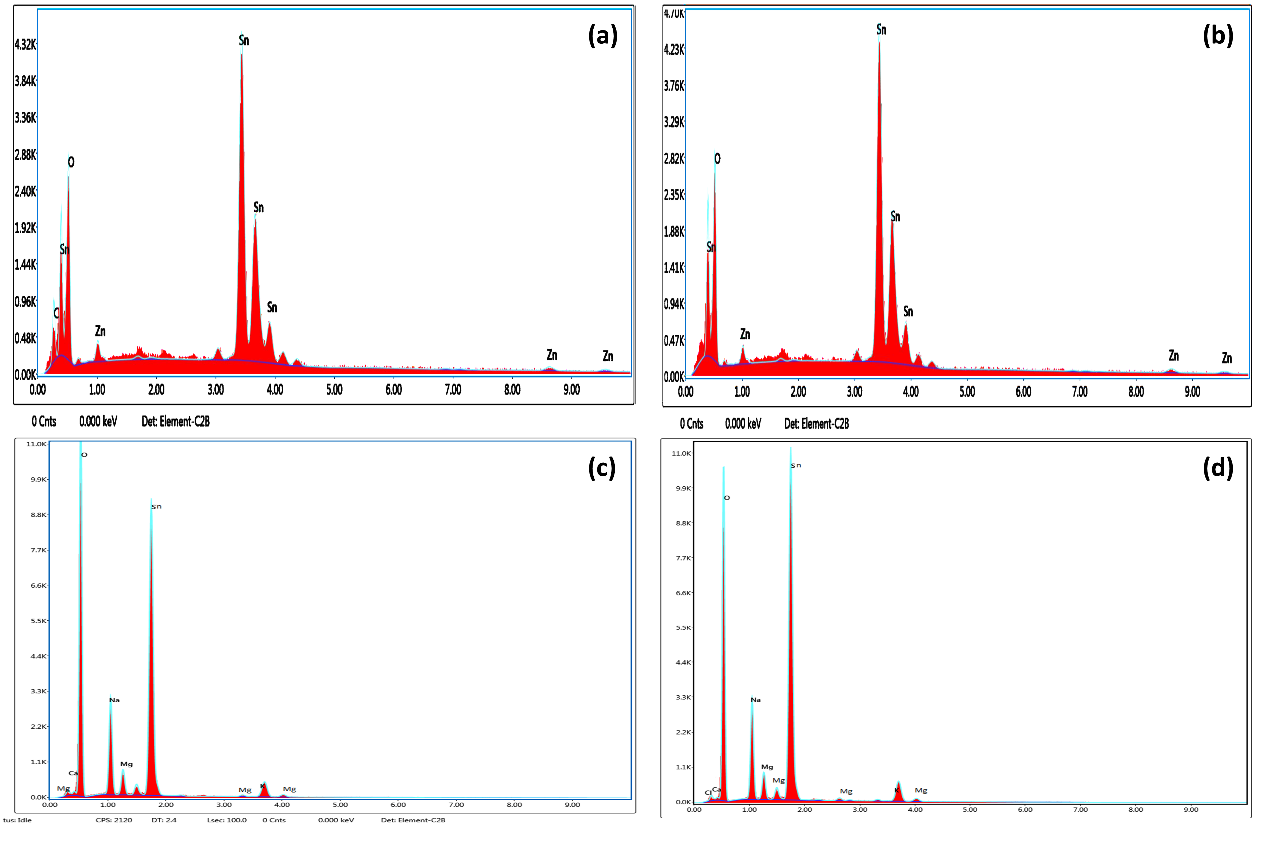


**Fig. 6S** EDAX analysis of ZTO thin film ((a) without and (b) annealing) and MTO thin film ((c) Without and (d) with annealing)
